# Therapeutic Redox Reprogramming in Breast Cancer: From Antioxidant Modulation to Precision Nanomedicine

**DOI:** 10.3390/antiox15101307

**Published:** 2026-10-10

**Authors:** Nina Kummer, Sabina Gayibova, Takhir Aripov, Désirée Gül, Mustafa Dolaz, Burcu Önal Acet, Leyla Polat Köse, Mehmet Odabaşı, Roland H. Stauber, Ömür Acet

**Affiliations:** 1 Nanobiomedicine/Molecular and Cellular Oncology, ENT, University Medical Center Mainz, Langenbeckstrasse 1, 55101 Mainz, Germany; kummnina@uni-mainz.de (N.K.); rstauber@uni-mainz.de (R.H.S.); 2 Laboratory of Plant Cytoprotectors, Institute of Bioorganic Chemistry Named After A. Sadykov, Mirzo Ulugbek, 83, Tashkent 100125, Uzbekistan; gayibova.sabina@gmail.com (S.G.); aripov.takhir@gmail.com (T.A.); 3 Department of Environmental Engineering, Faculty of Engineering, Kyrgyz-Turkish Manas University, Bishkek 720038, Kyrgyzstan; mustafa.dolaz@manas.edu.kg; 4 Department of Engineering Fundamental Sciences, Faculty of Engineering, Artvin Çoruh University, Artvin 08000, Türkiye; burcuonal@artvin.edu.tr; 5 Medicinal-Aromatic Plants Application and Research Center, Artvin Çoruh University, Artvin 08000, Türkiye; 6 Department of Pharmacy Services, Vocational School, İstanbul Beykent University, Istanbul 34500, Türkiye; leylakose@beykent.edu.tr; 7 Faculty of Arts and Science, Chemistry Department, Aksaray University, Aksaray 68100, Türkiye; modabasi@aksaray.edu.tr; 8 Vocational School of Health Science, Pharmacy Services Program, Tarsus University, Tarsus 33400, Türkiye

**Keywords:** breast cancer, redox homeostasis, therapeutic redox modulation, oxidative stress, antioxidant therapy

## Abstract

Breast cancer progression and therapeutic resistance are closely associated with disrupted redox homeostasis, in which oxidative stress drives tumor growth, metabolic adaptation, and treatment failure. Although antioxidants have traditionally been considered cytoprotective agents, their effects on tumor redox signaling are highly context-dependent, creating an antioxidant paradox that limits their therapeutic application. This review examines how dysregulated redox signaling shapes breast cancer biology and therapeutic response across ER+, HER2+, and triple-negative subtypes, with emphasis on the NRF2–KEAP1, p53, PI3K/AKT, MAPK, and HIF-1α pathways. These mechanisms provide a biological rationale for selectively modulating tumor redox states rather than broadly suppressing oxidative stress. Within this framework, redox-responsive nanocarriers and nanozymes offer opportunities for precise spatial and temporal redox modulation, enhanced tumor targeting, and controlled therapeutic activity while potentially minimizing effects on healthy tissues. Redox-directed nanotherapeutics may also influence the tumor immune microenvironment, angiogenesis, and metabolic rewiring. However, clinical translation remains constrained by dose- and context-dependent effects, intratumoral heterogeneity, limited biomarker validation, and inadequate predictive models. Personalized redox nanomedicine integrating subtype-specific redox profiling, smart nanotherapeutic platforms, and biomarker-guided patient stratification could establish redox modulation as a precision therapeutic axis and improve treatment outcomes in breast cancer.

## 1. Introduction

Breast cancer (BC) remains one of the major global health challenges and is the most commonly diagnosed cancer among women worldwide. Despite substantial advances in early detection and treatment, BC continues to contribute considerably to cancer-related morbidity and mortality, underscoring the need for a deeper understanding of the molecular mechanisms underlying disease progression and therapeutic resistance. Among its major molecular subtypes, triple-negative breast cancer (TNBC) is characterized by aggressive tumor biology, a higher risk of recurrence, and poorer clinical outcomes. These features highlight the substantial biological and therapeutic heterogeneity of BC and the need for more precise and effective treatment strategies. Conventional systemic therapies, including chemotherapy, remain important components of BC treatment but are frequently associated with treatment-related toxicities and other adverse effects. These limitations have increased interest in natural products and other bioactive compounds as potential sources of anticancer and redox-modulating strategies. Increasing evidence suggests that their therapeutic effects may extend beyond direct cytotoxicity and involve modulation of reactive oxygen species (ROS), antioxidant defenses, and redox-dependent signaling pathways [1,2].

Antioxidants are compounds that prevent or slow the oxidation of other molecules. Mechanistically, they function primarily as hydrogen or electron donors. The human body has evolved complex antioxidant defense systems that act synergistically to protect cells from free radical-induced damage by scavenging ROS, enhancing antioxidant defenses, maintaining mitochondrial function, and inhibiting oxidative processes through free radical neutralization. Antioxidants may be natural or synthetic and can be classified as enzymatic or non-enzymatic compounds. Examples include enzymatic antioxidants such as superoxide dismutase (SOD) and catalase (CAT), as well as dietary antioxidants like vitamin C, tocopherols, tocotrienols, carotenoids, and vitamin E [1].

Since the mid-20th century, antioxidants have been shown to reduce the incidence of various conditions, including aging, cancer, inflammation, and liver diseases. They are widely used as dietary supplements, preventive agents against several disorders, and supportive therapies to alleviate the adverse effects of chemotherapy and radiotherapy by lowering oxidative stress in cancer patients [2]. Moreover, antioxidants may help reduce the morbidity associated with chemotherapeutic agents due to their effectiveness in mitigating chemotherapy-induced toxicity, which may contribute to improved survival rates and enhanced therapeutic outcomes in cancer patients [3]. Nevertheless, the role of antioxidants in cancer treatment, particularly in breast carcinogenesis, remains controversial. This is largely because the same compound may exhibit either antioxidant or pro-oxidant activity depending on factors such as concentration, tumor cell type, timing, and environmental conditions [4]. In this context, the pro-oxidant effects of pharmacological ascorbate (vitamin C) have been investigated in rats using different administration routes (i.v., i.p., and oral) and clinically relevant doses. These studies demonstrated that pharmacological ascorbate acts as a prodrug, promoting the steady-state formation of ascorbate radicals and H_2_O_2_ in the extracellular space, but not in the bloodstream. Furthermore, daily administration of ascorbate in mouse models of pancreatic, ovarian, and glioblastoma tumors significantly suppressed tumor growth, suggesting that pharmacological ascorbate may function as a pro-oxidant prodrug with therapeutic potential in cancer treatment [5,6]. In BC, the antioxidant enzyme peroxiredoxin-1 (PRDX1) has emerged as a potential biomarker and therapeutic target. Downregulation of PRDX1 significantly reduced BC cell proliferation and tumor growth in xenograft models using PRDX1-deficient MCF-7 cells. Additionally, in triple-negative breast cancer (TNBC), PRDX1 plays a critical role in maintaining redox balance and exerting cytoprotective effects. Consistently, PRDX1 knock down inhibited cell proliferation in vitro, and these inhibitory effects were further enhanced when combined with pro-oxidant agents such as H_2_O_2_, glucose oxidase, sodium L-ascorbate, or menadione [7].

Redox homeostasis represents an important biological determinant of BC behavior and therapeutic response. However, the redox landscape of BC is not uniform across molecular subtypes. ER+ tumors exhibit redox regulation that is closely associated with estrogen signaling, cellular metabolism, and antioxidant defense, whereas HER2+ tumors may display increased reactive oxygen species generation as a consequence of enhanced proliferative and metabolic signaling. These differences can shape the cellular response to oxidative stress and influence tumor progression, treatment sensitivity, and the development of therapeutic resistance. Therefore, understanding BC through a subtype-specific redox perspective is essential, particularly when considering the distinct biological characteristics of ER+, HER2+, and triple-negative BCs [8].

The evolution of the antioxidant paradigm in BC represents a significant shift in oncology, transitioning from the traditional perspective that antioxidants simply protect against carcinogenic damage to a more advanced understanding that cancer cells can actively reprogram their antioxidant defense systems to tolerate oxidative stress and acquire resistance to therapy [9,10,11]. In contemporary oncology research, attention has shifted from the conventional protective role of antioxidants to understanding and targeting the mechanisms by which BC cells dynamically reprogram their redox homeostasis. This adaptive process includes profound metabolic and transcriptional alterations that allow tumor cells to tolerate oxidative stress, maintain survival, and enhance therapeutic resistance [9].

The literature relevant to this article was identified through targeted searches in the PubMed/MEDLINE, Scopus, and Web of Science databases, focusing primarily on publications from the 2016–2026 period. The search was conducted in 2026, and studies were selected based on their relevance to the scope of the review, with priority given to recent, mechanistically informative, clinically relevant, and seminal studies. The searches covered topics such as breast cancer, reactive oxygen species, redox homeostasis, oxidative stress, antioxidants, redox signaling, treatment resistance, the tumor microenvironment, nanomedicine, nanozymes, and redox-responsive drug delivery. The identified studies were prioritized based on their direct relevance to the scope of this review, mechanistic contributions, methodological suitability, therapeutic significance, and levels of preclinical or translational evidence. While preclinical evidence was evaluated primarily in terms of mechanistic findings and proof-of-concept data, greater weight was placed on clinical evidence when addressing translational relevance, safety, biomarkers, and therapeutic applicability. Groundbreaking studies that established fundamental concepts or provided necessary mechanistic context were also considered where appropriate. Studies were selected based on their relevance and alignment with the review’s main themes rather than within the framework of a systematic review or meta-analysis. In this context, the review examines the emerging role of antioxidants not merely as protective agents against oxidative damage, but as active and context-dependent modulators of breast cancer redox homeostasis. Particular emphasis is placed on the molecular mechanisms underlying redox reprogramming across different breast cancer subtypes and the impact of antioxidant-mediated alterations in redox signaling on tumor progression, metabolic adaptation, tumor microenvironment interactions, and treatment resistance. This review also evaluates nanotechnology-based antioxidant delivery systems—including nanozymes and redox-responsive platforms—highlighting their potential to enhance targeting efficiency, controlled redox modulation, and therapeutic viability. The distinctive perspective of this review lies in its integration of antioxidant biology, redox-driven tumor adaptation, and nanomedicine within a holistic translational framework. Rather than focusing primarily on the cytoprotective effects of antioxidants, we emphasize their dual and context-dependent roles in shaping cellular signaling networks and treatment responses, and discuss how precisely controlled redox modulation via advanced nanoplatforms can contribute to more effective and personalized breast cancer therapy.

## 2. Redox Homeostasis in Breast Cancer Biology

### 2.1. Sources of Reactive Oxygen Species (ROS) in Breast Tumors

In BCs, ROS arise from both intracellular and microenvironmental sources and influence disease progression and therapy response.

#### 2.1.1. Mitochondrial ROS (mtROS)

In BC cells, mtROS production increases when electron transport through complexes I and III becomes inefficient, particularly under conditions of metabolic rewiring, enhanced fatty-acid oxidation (FAO), or altered mitochondrial dynamics (fission/fusion). In vivo, aggressive and metastatic BC cells display elevated mtROS levels and fragmented mitochondrial networks [12,13].

• Inhibition of the mitochondrial fission regulator DRP1 suppressed brain metastatic burden in HER2+ and brain-tropic models. Genetic depletion or pharmacologic inhibition of DRP1 resulted in a significant reduction in ex vivo brain photon flux and in the number of GFP+ metastatic lesions per brain (*p* < 0.01). Pharmacological inhibition of DRP1 also attenuated established brain metastases in orthotopic models, indicating that mitochondrial plasticity, FAO, and redox homeostasis jointly contribute to metastatic colonization [12].
• Treatment with the mitochondria-targeted antioxidant MitoQ reduced metastatic colonization and prevented recurrence in TNBC models. In the spontaneous MMTV-PyMT model, MitoQ treatment also improved recurrence-free survival following primary tumor resection [14].
• Induction of mitochondrial fusion was similarly associated with lower metastatic competence. Increased mitochondrial elongation decreased the number of metastatic lesions and the incidence of overt metastases in orthotopic and experimental metastasis models [13].

Taken together, these observations indicate that mitochondrial dynamics and redox regulation are closely interconnected determinants of metastatic competence, rather than isolated features of tumor metabolism. However, mitochondrial ROS homeostasis is also shaped by extramitochondrial sources that actively regulate oxidative signaling in breast cancer cells. Among these, NADPH oxidases represent major enzymatic sources of regulated ROS production and therefore provide an additional mechanistic link between redox signaling and tumor progression.

#### 2.1.2. NADPH Oxidases (NOX Family)

NOX enzymes generate extracellular or membrane-associated ROS and are expressed not only in cancer cells but also in cancer-associated fibroblasts (CAFs) and infiltrating myeloid cells. Among the NOX isoforms, NOX4 has been most consistently linked to stromal remodelling in mammary tumors, whereas NOX2 appears to play a similar role within immune compartments. One proposed mechanism is oxidation of PTEN followed by activation of PI3K/AKT signaling, which supports invasive behavior as well as vascular and lymphatic remodelling [15]. Both genetic depletion and pharmacological inhibition of this enzyme in BC models reduce invasive stromal phenotypes and tumor growth in vivo and enhance responses to systemic therapies. NOX4 knockdown or small-molecule inhibition reduced orthotopic lesion growth, invasion indices, and microvascular and lymphatic markers in treated mice, including invasive index and vessel density [16]. NOX-targeted strategies reduce ROS signaling and thereby limit stromal remodelling and metastasis. Isoform selectivity and compartment-specific targeting (tumor versus stromal cells) are required to preserve ROS-dependent antimicrobial and immune functions [15,16].

#### 2.1.3. Tumor Microenvironment (TME)

The tumor microenvironment (TME) provides an additional source of ROS in BCs. Abnormal perfusion, inflammatory cell activity, and repeated cycles of hypoxia followed by reoxygenation all contribute to oxidative stress within the local microenvironment. These conditions transiently increase ROS levels and can also leave a lasting imprint on cellular behavior. Intratumoral hypoxia provides a particularly clear example. In orthotopic BC models, hypoxia increased lung metastasis formation by approximately fivefold. Post-hypoxic cells retained HIF- and NF-κB-associated transcriptional programs and remained more resistant to oxidative stress after reoxygenation. In experimental metastasis assays, this population showed a markedly higher probability of metastatic outgrowth, with an estimated ~5-fold relative risk for lung metastasis within the fate-mapped compartment [17]. Consistent with this, murine mammary tumor models show that loss of antioxidant enzymes, including GPx2, raises intratumoral ROS, activates HIF-1α signaling, and promotes metastatic progression in vivo [15]. Oxidative stress in the TME can induce durable adaptations that persist after dissemination and continue to influence tumor-cell behavior beyond the primary site. Strategies aimed at reshaping the niche, such as vascular normalization, inhibition of HIF signaling, or modulation of CAF-associated redox programs, may be more promising than indiscriminate systemic antioxidant supplementation. This strategy is not without risk, however, because it could also shield disseminated tumor cells from ROS-dependent constraints during dissemination and early colonization [15,17,18]. ROS in BC do not behave as a single homogeneous entity. Mitochondrial ROS, NOX-derived ROS, and ROS generated within the microenvironment act through partly distinct signaling networks and can influence different stages of disease progression. This compartment-specific view helps explain why ROS-targeted interventions may produce different outcomes depending on where and when they act.

### 2.2. Redox Imbalance Across Breast Cancer Subtypes

The distribution and functional importance of ROS differ markedly across molecular subtypes, and recent in vivo work has clarified distinct vulnerabilities in each setting.

#### 2.2.1. ER-Positive (ER+) Tumors

ER+ (luminal) tumors show moderate basal ROS and relatively strong antioxidant buffering in vivo and in cell line xenografts. SLC7A11- and ferroptosis-related pathways are variably engaged in this subtype, but available preclinical data suggest that they remain therapeutically tractable. This variability stems from heterogeneous SLC7A11 (xCT) expression across ER+ tumors: SLC7A11-high tumors import more cystine and sustain higher glutathione synthesis, raising the threshold for ferroptotic death and favoring a more treatment-resistant phenotype, whereas SLC7A11-low tumors are comparatively ferroptosis-sensitive; this expression gradient, rather than a uniform subtype-wide effect, accounts for the divergent outcomes reported across studies. In luminal models, inhibition of SLC7A11, achieved either genetically or pharmacologically, enhances the efficacy of CDK4/6 inhibitors and results in greater tumor growth suppression. Combined treatment leads to significantly greater reductions in tumor volume than either therapy administered alone [19]. In ER+ disease, a more plausible clinical strategy is selective perturbation of antioxidant pathways, such as the cystine import/SLC7A11 axis, to sensitize tumors to standard therapy rather than broadly increasing ROS.

#### 2.2.2. HER2-Positive (HER2+) Tumors

HER2 amplification increases metabolic flux and frequently elevates ROS production in orthotopic HER2+ models. Studies of brain metastasis indicate that HER2+ lesions rely on FAO and mitochondrial plasticity to maintain redox homeostasis. Inhibiting FA synthesis or mitochondrial plasticity markedly suppresses the growth of brain metastases [12,15]. These observations provide a rationale for combining HER2-directed therapy with redox-modulating approaches, including FASN inhibition and DRP1 modulation, particularly in metastasis-focused settings.

The clinical relevance of HER2 biology has broadened with the emergence of HER2-low disease (immunohistochemistry [IHC] 1+, or IHC 2+ with a negative in situ hybridization result), which comprises roughly half of tumors previously classified simply as HER2-negative and is now a therapeutic target in its own right following the approval of the antibody-drug conjugate trastuzumab deruxtecan on the basis of the DESTINY-Breast04 trial [20]. Redox biology in HER2-low tumors is not simply a scaled-down version of classic HER2-overexpressing disease: optical redox-ratio measurements place ER+/HER2-negative cells between TNBC (lowest ratio) and HER2-overexpressing cells (highest ratio) [21], and HER2-overexpressing tumors show lower serum antioxidant capacity than HER2-negative disease [22], consistent with an intermediate, receptor-density-dependent redox phenotype rather than a binary HER2-positive/negative split. Because HER2-low tumors express insufficient receptor density to drive the FAO- and mitochondrial-plasticity-dependent phenotype described above for HER2-amplified disease, but retain enough HER2 expression to be targeted by antibody-drug conjugates, their redox vulnerabilities are likely governed more by hormone-receptor co-expression and payload-induced oxidative/DNA-damage stress than by HER2-driven mitochondrial rewiring itself. This distinction has direct therapeutic relevance: redox-modulating strategies developed for HER2-amplified disease should not be assumed to translate directly to HER2-low tumors, and dedicated studies of redox phenotypes across the full HER2 expression continuum (negative, low, and amplified) are needed.

#### 2.2.3. Triple-Negative Breast Cancer (TNBC)

TNBC (basal-like) displays the most pronounced oxidative imbalance. Basal mtROS levels are elevated across multiple TNBC cell lines and orthotopic models, and several in vivo studies demonstrate that TNBC metastasis is highly sensitive to mitochondrial ROS modulation. MitoQ decreased lung metastasis counts in MDA-MB-231 tail-vein and spontaneous metastasis models and improved recurrence-free survival after surgery [14]. Enforced mitochondrial fusion or DRP1 inhibition reduced metastatic seeding and overt metastases in TNBC and brain-tropic models [12,13]. TNBC subtypes differ in ferroptosis sensitivity, with LAR tumors showing increased susceptibility. Ferroptosis inducers reduce tumor burden in responsive xenograft and PDX models [23]. A distinct basal-cell mitochondrial ROS mechanism also contributes to chemoresistance: a C15ORF48-driven basal subpopulation with altered mitochondrial ROS regulation persists after anthracycline-based chemotherapy in TNBC patient-derived xenografts, and its knockdown restores chemosensitivity by increasing ROS and apoptosis, identifying mitochondrial ROS control as a residual-disease vulnerability distinct from the mtROS-suppression strategies described above [24]. Consistent with a therapy-timing-dependent role for mtROS, sublethal doxorubicin exposure has also been shown to induce mitochondrial ROS that promotes TNBC cell migration and invasion, an effect blocked by the mitochondria-targeted antioxidant MitoTEMPO in preclinical models [25]. Together, mtROS dependence and heterogeneous ferroptosis sensitivity point to two distinct therapeutic avenues in TNBC: suppression of compartmentalized mtROS to limit dissemination and recurrence, and pro-oxidant or ferroptosis-inducing strategies in molecularly susceptible subsets. In both settings, timing relative to cytotoxic therapy is likely to be critical.

**Figure 1:**
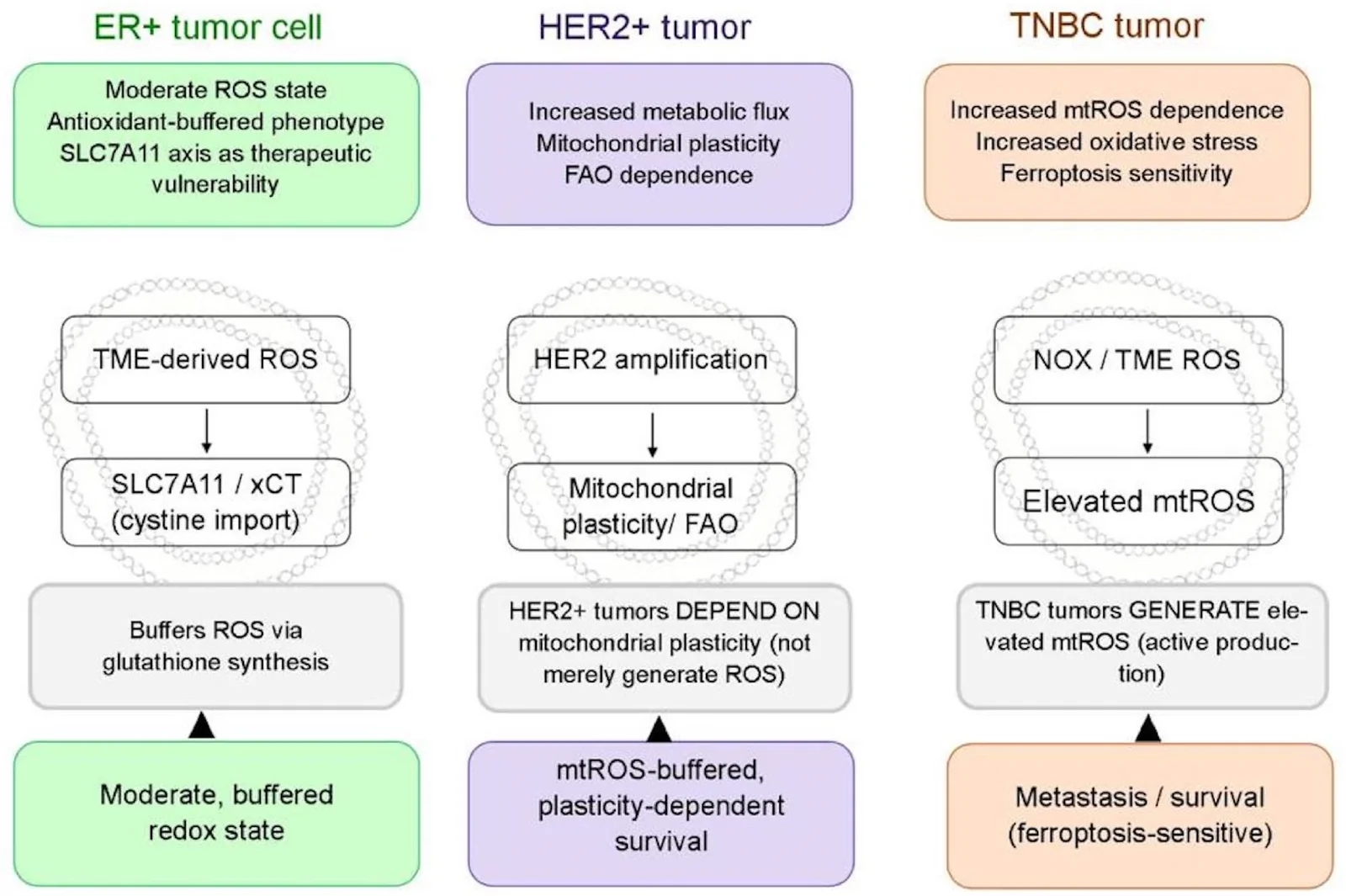
Compartmentalized redox signaling and subtype-specific vulnerabilities in breast cancer. Each column is read from top to bottom: the coloured box summarizes the subtype redox phenotype; the dotted outline denotes the tumor cell; solid arrows indicate the direction of a causal, experimentally supported relationship (upstream event → downstream redox consequence); the grey box states the interpretation drawn in this review; and the triangular arrowhead links it to the resulting net phenotype below. Arrows encode direction only, not flux magnitude or pathway exclusivity, and the three configurations are not mutually exclusive in heterogeneous tumors. Demonstrated in breast cancer models: FAO- and mitochondrial-plasticity dependence of HER2+ brain metastases, suppressed by DRP1 inhibition; elevated mitochondrial ROS in TNBC, with MitoQ reducing lung metastasis and improving recurrence-free survival and with mitochondrial fusion reducing metastatic seeding; LAR-enriched ferroptosis sensitivity; and SLC7A11 blockade enhancing CDK4/6 inhibition in luminal models. Proposed rather than demonstrated: TME-derived ROS as the upstream input to SLC7A11 in ER+ tumors, and NOX-derived ROS as a driver of the elevated mitochondrial ROS state in TNBC; both integrate separate lines of evidence and have not been directly tested. The HER2+ panel depicts HER2-amplified disease only.

Overall, BC redox biology is better understood as a compartment-resolved, subtype-specific process than as generalized oxidative stress. This framework helps explain why mtROS, NOX-derived ROS, and microenvironmental ROS differ in their effects on metastasis and therapy response, and why selective redox targeting is likely to be more informative therapeutically (Table 1). Compartmentalized redox signaling and subtype-specific vulnerabilities in BC shown in Figure 1.

## 3. Molecular Basis of Redox Reprogramming

Reactive oxygen species function as central signaling mediators regulating multiple oncogenic and stress-responsive pathways in BC, including NRF2-dependent antioxidant responses, p53 signaling, hypoxia adaptation, and PI3K/AKT-mediated metabolic reprogramming. These redox-oncogenic connections follow a broadly conserved sequence.

(i) ROS oxidize reactive cysteine thiols on upstream sensors and phosphatases, including KEAP1, PTEN, and other protein tyrosine phosphatases, altering their conformation, activity, or stability; reversible cysteine sulfenylation (formation of disulfides or sulfenic acid) is the direct chemical event, with irreversible sulfinylation/sulfonylation occurring under sustained, higher-intensity stress.
(ii) This releases or activates downstream transcriptional effectors, principally NRF2, HIF-1α, NF-κB, and AP-1, and kinase cascades, principally PI3K/AKT and RAF/MEK/ERK.
(iii) These effectors reprogram transcriptional and metabolic machinery, including antioxidant gene batteries (glutathione and thioredoxin pathway enzymes), glycolytic and pentose-phosphate-pathway enzymes, and epithelial–mesenchymal transition regulators.
(iv) The resulting metabolic and transcriptional state feeds back on ROS production itself, for example via NADPH output or mitochondrial dynamics, generating the self-reinforcing circuits described in each subsection below. This cysteine-oxidation-centered framework, rather than a diffuse notion of generalized “oxidative damage,” is the mechanistic thread that connects the four pathways discussed in this section.

### 3.1. NRF2-KEAP1 Signaling Axis

NRF2 functions as a key regulator of antioxidant response elements. While transient activation preserves cellular redox balance, sustained NRF2 activity contributes to chemoresistance and metabolic reprogramming across cancers. Qin et al. [27] reported that ionizing radiation (IR) induces NRF2 activation in TNBC cells. Genetic or pharmacologic inhibition of NRF2 (siRNA or ML385) increased IR-induced ROS accumulation, impaired DNA-PK-dependent repair of DNA double-strand breaks, reduced clonogenic survival, and decreased cancer stem cell (CSC) marker expression in TNBC models. NRF2 activation therefore supports the maintenance of therapy-resistant cancer stem-like populations. These findings identify NRF2 as a liability in therapy-resistant subpopulations rather than a uniformly cytoprotective factor.

This NRF2–radioresistance link was defined in TNBC, but NRF2-driven therapy resistance is not confined to basal-like disease, and within each of the other two subtypes it operates through more than one distinct route. In ER+ breast cancer, two separate mechanisms converge on NRF2: tamoxifen exposure itself increases NRF2 activity and antioxidant gene expression in both cultured cells and patient tumor tissue obtained before and after short-term neoadjuvant tamoxifen, underlying cross-resistance of tamoxifen-resistant cells to subsequent irradiation, with pharmacological NRF2 inhibition (ML385) re-sensitizing these cells to radiation-induced killing [28,29]; independently, FGFR2 signaling induces AMPKα/ULK1-dependent autophagy, and the autophagy receptor p62/SQSTM1 then promotes dissociation of the KEAP1–NRF2 complex, activating NRF2 and blunting the response of ER+ cells to anti-estrogen drugs such as tamoxifen and fulvestrant, with high NFE2L2 (the NRF2 gene) expression carrying independent prognostic value in this setting. In HER2+ disease, NRF2 likewise acts through two separable mechanisms: it is dispensable in the primary tumor but becomes selectively essential after HER2-targeted therapy is withdrawn, when HER2 downregulation triggers an NRF2-dependent metabolic reprogramming (restoring redox homeostasis and upregulating de novo nucleotide synthesis) that is both necessary and sufficient for recurrence of dormant tumor cells, with NRF2-high recurrent tumors becoming newly sensitive to glutaminase inhibition [30]; separately from this recurrence-linked route, active HER2 also binds NRF2 directly in the primary tumor setting, increasing NRF2-dependent antioxidant-response-element transcription and upregulating drug-resistance genes (HO-1, MRP5, GSTA2, GSTP1, CYP3A4) to confer chemotherapy resistance [28]. Neither the Kang nor the Górska-Arcisz [28,31] study frames its mechanism in these terms, but considered together they suggest a working distinction: the direct HER2–NRF2 binding described above appears to bypass the canonical KEAP1 checkpoint, whereas the ER+ autophagy mechanism still operates through KEAP1, via autophagy-driven dissociation of the KEAP1–NRF2 complex. We propose this KEAP1-independent versus KEAP1-dependent framing as an interpretation that follows from combining the two datasets, rather than as a conclusion either paper draws itself. Together, these five studies indicate that NRF2 supports therapy resistance and recurrence through distinct, subtype- and context-specific downstream programs—DNA-repair support and cancer-stem-cell maintenance in TNBC; endocrine- and radiation-cross-resistance together with autophagy-linked KEAP1 dissociation in ER+ disease; and recurrence-enabling nucleotide/redox metabolism together with direct receptor-level, KEAP1-independent activation in HER2+ disease—rather than a single uniform mechanism across subtypes.

NRF2 activation increases flux through glutathione biosynthesis and the pentose phosphate pathway, expands NADPH and GSH pools, and raises the threshold for ROS-mediated cytotoxicity. In doing so, it enables subclones to withstand pro-oxidant stress while sustaining anabolic growth [16]. These metabolic relationships place NRF2 at the intersection of redox buffering, anabolic metabolism, and DNA repair in breast tumors.

### 3.2. p53 and Redox Regulation

p53 is a central stress-responsive transcription factor whose functions extend beyond classical tumor suppression. In its wild-type form, p53 acts as a bidirectional regulator of cellular responses to oxidative stress. Under moderate or transient ROS levels, it activates antioxidant programs that preserve genomic integrity. These include SESN1/2, TIGAR, GPX1, and enzymes involved in glutathione homeostasis. The net effect is lower intracellular ROS, less oxidative DNA damage, and preservation of mitochondrial function.

When oxidative stress becomes sustained or severe, p53 shifts toward a pro-oxidant and pro-apoptotic program. Induction of PUMA, NOXA, and BAX, together with repression of antioxidant genes, promotes ROS accumulation, mitochondrial outer membrane permeabilization, and apoptosis. Thus, p53 functions as a rheostat that couples stress intensity to cell fate.

TP53 mutations occur in approximately 30–40% of BCs and in most cases of TNBC. Earlier studies focused on genomic instability and cell-cycle control, whereas more recent work indicates that p53 status also governs adaptation to metabolic and oxidative stress at both cellular and microenvironmental levels.

Loss of p53 reshapes the TME and promotes pro-metastatic signaling. In mammary tumor models, p53 deficiency increases systemic inflammation and recruits neutrophils that facilitate metastatic dissemination. Neutrophil extracellular traps (NETs) can reactivate dormant tumor cells in the lung and promote metastatic outgrowth [18]. The result is a more inflammatory, stress-tolerant microenvironment that supports survival and metastatic progression.

Mutant p53 further contributes through active metabolic reprogramming. Gain-of-function variants enhance glycolysis, alter mitochondrial function, and increase reliance on NADPH-generating pathways. As a result, antioxidant capacity expands and the threshold for cytotoxic stress rises. Tumor cells maintain proliferative signaling while tolerating both endogenous and therapy-induced stress. In this context, mutant p53 sustains a controlled intracellular state that enables survival under otherwise lethal conditions.

p53 status also determines ferroptosis sensitivity. Wild-type p53 represses SLC7A11, limits cystine import, reduces glutathione synthesis, and promotes lipid peroxidation, thereby increasing susceptibility to ferroptotic cell death. Certain TP53 mutations attenuate this response and confer resistance to ferroptosis [17]. Such differences define mutation-specific vulnerabilities with therapeutic relevance.

p53 in BC functions not only as a guardian of genome integrity but also as a regulator of redox adaptation, ferroptosis sensitivity, and microenvironmental signaling. These roles make stress-adaptation pathways, particularly ferroptosis control, antioxidant buffering, and mutant p53-associated metabolic programs, attractive therapeutic targets in p53-altered disease.

### 3.3. PI3K/AKT and MAPK Pathways

Mitochondrial ROS influence major oncogenic signaling pathways in BC, particularly the PI3K/AKT and MAPK cascades [32]. Early biochemical studies showed that ROS can reversibly oxidize catalytic cysteine residues in protein tyrosine phosphatases such as PTEN. This inhibits phosphatase activity and sustains PI3K/AKT signaling [33]. ROS also influence multiple components of the MAPK cascade, shaping ERK and p38 activity through modulation of upstream kinases and phosphatases.

Activation of PI3K/AKT signaling, in turn, alters cellular metabolism in ways that reinforce antioxidant capacity. Increased glucose uptake and enhanced flux through the pentose phosphate pathway elevate NADPH production, supporting intracellular buffering systems [34]. What remained less clear until recently was whether these biochemical relationships directly shape metastatic behavior in vivo.

Studies in TNBC models demonstrate that mitochondrial dynamics directly influence signaling activity. Modulation of DRP1-dependent fission or induction of mitochondrial fusion alters ROS production and distribution, which affects AKT and ERK phosphorylation. In these models, experimental manipulation of mitochondrial dynamics altered ROS distribution, AKT/ERK signaling, migration, and metastatic behavior, indicating a direct functional link between mitochondrial morphology and oncogenic signaling output. These findings argue that mitochondrial ROS act as direct regulators of signaling output rather than as passive metabolic by-products [13].

Redox-dependent feedback loops further stabilize oncogenic signaling. Oxidative inactivation of PTEN sustains PI3K/AKT activation even after transient stress. Activated AKT promotes anabolic metabolism and increases NADPH production through the pentose phosphate pathway and malic enzyme activity. NRF2 activation further strengthens antioxidant systems, including glutathione and thioredoxin pathways. A self-reinforcing circuit therefore emerges, in which transient oxidative signals give rise to durable changes in signaling and metabolism.

In this framework, mitochondrial dynamics, phosphatase oxidation, and metabolic rewiring act together to stabilize PI3K/AKT and MAPK activity under stress. Such a model also points to therapeutic opportunities: disrupting mitochondrial morphology, NADPH supply, or PTEN redox control may destabilize signaling states that sustain metastasis and therapy resistance.

This discussion of PTEN oxidation and membrane-associated PI3K/AKT signaling has so far omitted the structural organizer proteins that physically position PTEN, HER2, and Wnt receptors at the plasma membrane. NHERF1 (EBP50) and its paralog NHERF2 (E3KARP) are PDZ-domain scaffolding proteins that assemble macromolecular signaling complexes at the apical membrane of mammary epithelial cells. NHERF1 binds PTEN directly through its first PDZ domain and is required for PTEN membrane recruitment: NHERF1 loss delays turnover of PDGF-induced phospho-AKT in breast cancer cells and elevates phospho-AKT in the mammary glands of NHERF1-knockout mice, indicating that NHERF1 normally restrains PI3K/AKT signaling by keeping PTEN positioned at the membrane where oxidation-sensitive phosphatase activity is locally regulated [35]. NHERF1 also binds Frizzled receptors directly and restrains canonical Wnt/β-catenin signaling; NHERF1 loss in breast cancer cell lines and in knockout mouse mammary glands increases nuclear β-catenin activity and Wnt-dependent proliferation, and NHERF1 expression correlates negatively with nuclear β-catenin in human breast tumors [36]. At the HER2 locus specifically, NHERF1 forms a membrane complex with Ezrin and the LAP-family scaffold Erbin that stabilizes HER2/HSP90 interactions and retains active HER2 at the plasma membrane rather than allowing its internalization and degradation; loss of apical polarity during HER2-driven transformation redistributes this complex, and disrupting it destabilizes HER2 membrane retention [37]. Together, these scaffolding interactions indicate that NHERF1/NHERF2 spatially organize the same membrane compartment in which ROS-sensitive PTEN oxidation, HER2 trafficking, and Wnt/β-catenin signaling converge, and that loss or mislocalization of these organizer proteins can independently license sustained PI3K/AKT and Wnt output at the plasma membrane.

### 3.4. HIF-1α and Hypoxia-Induced Redox Adaptation

HIF-1α activation under hypoxic conditions has long been recognized as a central driver of glycolytic reprogramming and angiogenesis in cancer. Reduced oxygen availability inhibits prolyl hydroxylase activity, leading to HIF-1α stabilization and transcription of genes involved in glucose uptake, glycolysis, and vascular endothelial growth factor (VEGFA)-mediated angiogenesis. Although the link between hypoxia and ROS is well established, it has been less clear whether transient hypoxic exposure leaves durable phenotypic traces in BC cells.

**Figure 2:**
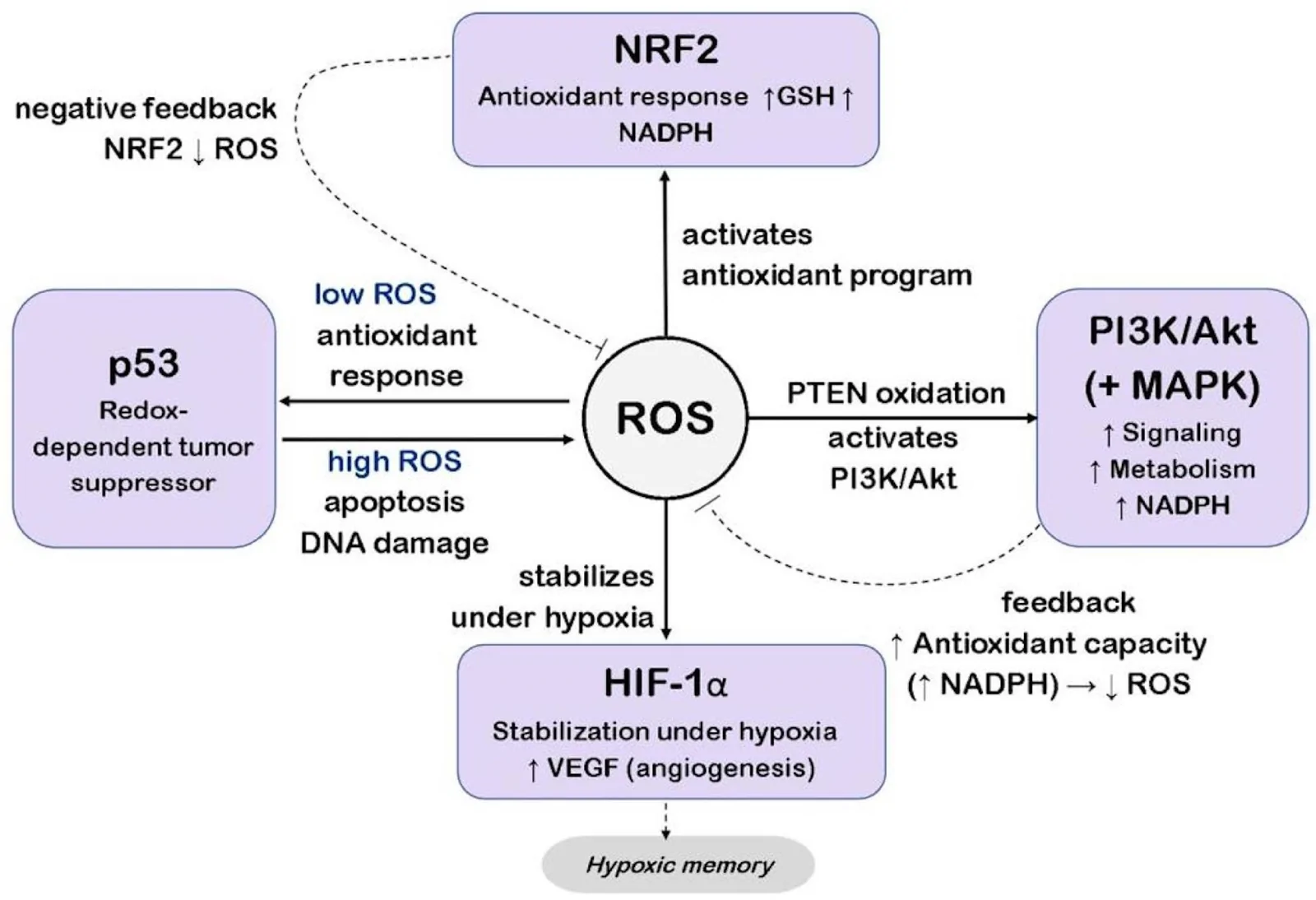
Integration of redox signaling pathways in breast cancer. ROS (central node) act as a shared second messenger converging on NRF2, p53, PI3K/AKT (with MAPK) and HIF-1α. Symbol key: solid arrows indicate a directional, experimentally supported input from ROS, with adjacent text specifying the proposed mechanism (e.g., PTEN oxidation); dashed arrows indicate feedback onto ROS levels rather than a primary signaling input; a dashed line ending in a blunt bar (⊣) denotes inhibitory negative feedback; upward arrows (↑) denote an increase; downward arrows (↓) denote a decrease; blue labels (“low ROS”, “high ROS”) mark threshold-dependent branching, in which the same node yields opposite outputs; and the grey ellipse denotes a persistent cellular state rather than a molecule. Arrows encode direction, not magnitude or kinetics. Demonstrated in breast cancer: NRF2-dependent radioresistance in TNBC, reversed by NRF2 inhibition; mutant-p53 redox rewiring and p53-dependent ferroptosis via SLC7A11; mitochondrial- and NOX4-dependent PI3K/AKT activation; and oxygen-independent HIF-1α stabilization after GPx2 loss, alongside post-hypoxic “hypoxic memory”. Proposed rather than demonstrated: the two dashed feedback loops, whose individual steps are supported but whose closed-loop behaviour has not been measured as depicted, and the low-/high-ROS p53 threshold, for which breast-tumor thresholds are undefined.

Transient hypoxic exposure generates persistent redox-adapted phenotypes associated with increased metastatic potential in BC. Fate-mapping experiments demonstrated that tumor cells exposed to intratumoral hypoxia acquire a long-lived, ROS-resistant state that persists after reoxygenation. Godet et al. [18] showed that post-hypoxic BC cells display increased survival during circulation and a significantly higher capacity to seed lung metastases compared with non-hypoxic counterparts. Follow-up studies, including Rocha et al. [26] and subsequent work by Godet et al. [15], confirmed that hypoxia-exposed cells retain altered transcriptional and metabolic programs consistent with enhanced oxidative stress tolerance and invasive competence even after oxygen levels normalize [26]. In summary, available evidence supports the concept of hypoxic memory, whereby transient hypoxic episodes generate stable, redox-tolerant states that contribute disproportionately to metastatic dissemination. Complementary work indicates that redox perturbation can also act upstream of hypoxia signaling and vascular dysfunction. In mammary tumor models, loss of glutathione peroxidase 2 (GPx2) increases intracellular ROS and stabilizes HIF-1α independently of classical oxygen deprivation. Ren et al. [16] linked GPx2 deficiency to increased ROS, enhanced VEGFA signaling, vascular abnormalization, and accelerated metastatic progression in vivo. Reversal of this phenotype by HIF-1α inhibition or restoration of GPx2 expression indicates that redox imbalance can initiate hypoxia-like signaling cascades that subsequently promote malignancy. Taken together, the findings challenge a purely oxygen-centric view of HIF biology and show that oxidative stress can itself induce hypoxic transcriptional programs and vascular dysfunction. HIF-1α therefore emerges not simply as a mediator of reversible oxygen deprivation, but as a node through which hypoxia and redox imbalance can generate durable transcriptional and metabolic states linked to metastatic fitness and therapy tolerance. Integration of redox signaling pathways in BC shown in Figure 2. Redox-regulated signaling pathways in BC was shown in Table 2.

## 4. Antioxidants as Redox-Reprogramming Agents

In BC, antioxidants act not only as radical scavengers but also as modulators of intracellular redox state, metabolic flux, and signaling output. Biological outcomes are determined by compound identity, subcellular targeting (e.g., mitochondrial vs. cytosolic), formulation (free vs. nano-delivery), and dose/timing: mitochondria-targeted antioxidants suppress metastatic competence, whereas catalytic nanozymes locally increase ROS to induce cytotoxicity.

### 4.1. Natural Antioxidants

Two themes dominate recent work on natural antioxidants in BC: delivery and combinatorial use. A consistent finding across preclinical studies is that pharmacokinetics, rather than intrinsic biological inactivity, often limits the therapeutic potential of natural compounds.

Delivery and bioavailability have emerged as critical determinants of in vivo efficacy. Resveratrol, despite robust antiproliferative effects in vitro, has historically shown limited translational success due to poor solubility and rapid metabolism. However, when formulated in peptide-sucrose liposomes (PSL@RES), liposomal resveratrol showed markedly enhanced antitumor activity in orthotopic BC models compared with the free compound. In MCF-7 xenografts, PSL@RES inhibited tumor growth at doses as low as 5 mg·kg^−1^ and achieved complete tumor inhibition at 10 mg·kg^−1^ in mice. Mechanistically, the nanoformulation increased intratumoral p53 expression and activated downstream apoptotic signaling pathways while reducing systemic toxicity relative to unformulated resveratrol [38]. The improved activity of liposomal resveratrol suggests that the mismatch between in vitro potency and in vivo performance often reflects delivery constraints rather than lack of biological activity.

In parallel, growing evidence has examined synergistic phytochemical combinations designed to influence not only tumor cell redox balance but also the tumor immune microenvironment. A defined oral mixture of resveratrol, curcumin, and quercetin (RCQ) was shown to suppress tumor progression in the 4T1 syngeneic TNBC model. Treatment reduced tumor growth and significantly reshaped the immune landscape, increasing CD4^+^ and CD8^+^ T cell infiltration and promoting Th1 polarization while decreasing immunosuppressive signatures within the TME. The observed immune changes suggest that combined phytochemical regimens can modulate tumor–host interactions beyond simple ROS scavenging.

Some natural antioxidants also act as chemosensitizers in aggressive BC models. Curcumin and related polyphenols continue to show capacity to potentiate the efficacy of standard chemotherapeutics in TNBC systems. Preclinical studies report that curcumin co-treatment enhances the antitumor activity of platinum agents such as carboplatin in TNBC models, with increased apoptosis and modulation of redox-sensitive survival pathways observed in vivo [39]. These effects are associated with alterations in oxidative stress signaling and suppression of pro-survival pathways, suggesting that phytochemicals can shift the intracellular redox threshold in a manner that enhances cytotoxic response.

### 4.2. Synthetic Antioxidants

Synthetic antioxidants show dose-dependent and often paradoxical effects, with subcellular targeting emerging as a major determinant of outcome.

• Mitochondria-targeted antioxidants (MitoQ) reduce metastatic competence in vivo. In orthotopic MDA-MB-231 and spontaneous MMTV-PyMT mouse models, MitoQ (a triphenylphosphonium-conjugated ubiquinone) administered at clinically relevant doses prevented metastatic take and post-surgical recurrence without impairing standard chemotherapy activity in vitro. Mechanistically, MitoQ reduced mitochondrial superoxide signaling involved in the acquisition of metastatic stem-like traits, and in vivo strongly decreased lung metastases and recurrence incidence. These preclinical data position mitochondrial antioxidants as candidate metastasis-preventive agents in TNBC models. Importantly, quantitative tumor end-points (metastatic nodules, recurrence-free survival) were significantly improved in treated cohorts [14].
• Small nitroxide antioxidants (Tempol) show concentration-dependent metabolic and redox effects. Tempol inhibits mitochondrial oxygen consumption and glutamine metabolism in cancer cells and reduces tumor growth in vivo. Its effects are strongly concentration-dependent: lower doses are protective, whereas higher doses induce oxidative stress and apoptosis. Mechanistically, Tempol disrupted central carbon metabolism, including IDH1/IDH2 activity and TCA-cycle flux, linking redox cycling to metabolic collapse at higher doses [40].
• SOD-mimetic and mitochondria-directed agents (Mn porphyrins/Mito-antioxidants): Mn-based SOD mimetics and related mitochondria-directed agents remain mechanistically relevant but less well defined translationally. Their effects appear highly context-dependent, particularly in combination with chemotherapy, where cytoprotection and tumor protection may be difficult to separate [14,40].

### 4.3. Nanozymes

Nanozyme-based strategies have expanded rapidly in BC research over the last several years. Unlike classical antioxidants, these catalytic nanostructures are designed to manipulate redox chemistry in situ, either by generating ROS locally or by modulating oxygen and hydrogen peroxide within the TME to enhance treatment response. CeO_2_-based nanoenzymes have been developed for combined photothermal–chemodynamic therapy. Hollow mesoporous CeO_2_ nanoenzymes loaded with indocyanine green (ICG) highlighted pH-responsive Fenton-like catalytic activity, producing ROS selectively under acidic tumor conditions. When combined with 808-nm near-infrared photothermal irradiation, these nanoenzymes eradicated established breast tumors in mice in a preclinical model. The study reported tumor regression, biodistribution, and safety data that strengthen the case for further preclinical development [41]. This work exemplifies how catalytic ROS amplification can be spatially confined and externally triggered to maximize tumor-specific cytotoxicity.

Platinum-based nanozymes have also shown promising activity in BC models. A nuclease-mimetic platinum nanozyme identified tumor-targeted DNA platination combined with oxidative DNA cleavage, overcoming platinum resistance in cancer cells in vivo in murine proof-of-concept experiments [42]. In another study, Fe_3_O_4_/Pt nanozymes loaded with 5-fluorouracil exhibited peroxidase- and catalase-like activities, generating ROS while simultaneously delivering chemotherapy. In 4T1 breast tumor-bearing mice, this dual catalytic-chemotherapeutic system significantly reduced tumor burden compared with controls [43]. Bimetallic PtCo nanozymes coated with gemcitabine-hyaluronic acid polyethylene glycol were evaluated in 4T1 and TNBC models, where they generated oxygen and hydrogen peroxide to relieve tumor hypoxia and increase ROS-mediated cell death. These nanozymes showed potent antitumor activity in vivo, although long-term organ histology revealed toxicity concerns that warrant further optimization [44].

Ruthenium-based nanozymes have provided additional mechanistic insight into catalytic redox tuning. A study in Nature Communications showed that engineered lattice expansion of ruthenium nanocrystals, approximately six percent expansion, enhanced multiple enzyme-like activities, including peroxidase- and oxidase-mimetic functions. Under an applied triboelectric stimulation paradigm, these engineered nanocrystals produced an approximately threefold increase in ROS generation and achieved superior breast tumor control in mouse models. Density functional theory analyses linked d-band center shifts resulting from lattice expansion to increased catalytic rates, providing a structure-activity framework for rational nanozyme design [45].

Together, these reports shift the role of redox-active materials in oncology from conventional antioxidant support to catalytic redox engineering. The traditional “antioxidant” paradigm has expanded to encompass catalytic redox reprogrammers—nanozymes that either generate ROS locally to induce tumor cell death or catalytically modulate tumor microenvironmental oxygen and hydrogen peroxide levels to sensitize tumors to therapy. Importantly, several of these investigations include comprehensive in vivo efficacy and safety assessments in BC models, and an increasing proportion specifically target TNBC, reflecting the growing recognition of redox vulnerabilities in aggressive subtypes. Importantly, the therapeutic role of nanozymes should be considered within the broader context of redox modulation rather than being equated with conventional antioxidant strategies. While antioxidant interventions generally aim to restore or maintain redox balance by limiting excessive ROS accumulation, ROS-generating nanozymes can exploit the catalytic features of nanomaterials to shift this balance toward a more oxidizing state. From a therapeutic perspective, this represents a different redox-based strategy in which oxidative stress is not simply regarded as a pathological consequence to be alleviated, but as a potentially exploitable determinant of tumor-cell vulnerability. Depending on their catalytic properties and the biochemical characteristics of the tumor microenvironment, nanozymes may therefore modulate ROS-related reactions in ways that enhance oxidative pressure, alter local oxygen or hydrogen peroxide availability, and ultimately influence cancer-cell survival. This conceptual shift—from restoring redox equilibrium to therapeutically redirecting it—highlights the potential of nanozymes as catalytic tools for redox-based cancer intervention.

### 4.4. Dose- and Context-Dependent Effects

The biological effects of redox-targeted interventions depend less on whether an agent is labeled “antioxidant” and more on where, how, and at what dose it acts. One of the most important is subcellular localization. Mitochondria-directed antioxidants such as MitoQ reduce metastatic dissemination in TNBC models. In contrast, non-targeted antioxidants show weaker or inconsistent effects [14]. The contrast between mitochondrial and non-targeted antioxidants suggests that intracellular localization strongly influences therapeutic efficacy, as global antioxidant approaches may not affect the redox pathways that drive metastasis.

Formulation further modifies activity by changing exposure, tumor accumulation, and toxicity. Nano- and liposomal delivery systems improve the in vivo performance of phytochemicals such as resveratrol and curcumin. These formulations increase bioavailability, allow effective dosing, and alter toxicity profiles compared with free compounds [46]. As a result, compounds with limited activity in free form can become effective in vivo once delivery is optimized.

Dose adds a further layer of complexity because many redox-active compounds behave biphasically. For example, Tempol acts as an antioxidant at low concentrations but induces oxidative stress and apoptosis at higher doses. This shift reflects redox cycling and disruption of central metabolic pathways, including isocitrate dehydrogenase and the tricarboxylic acid cycle. These effects have been observed in both cell-based systems and xenograft models and highlight the need for careful dose selection [40].

Nanozymes extend this logic even further by functioning as catalytic regulators rather than conventional antioxidants. Depending on their design, they can either amplify ROS locally to induce tumor cell death or generate oxygen to improve the efficacy of other therapies. Some increase ROS levels through peroxidase- or Fenton-like reactions to induce tumor cell death. Others generate oxygen through catalase-like activity and improve responses to chemotherapy or photodynamic therapy. Studies in TNBC models demonstrate substantial antitumor effects. However, potential systemic toxicity and long-term organ effects remain unresolved and require further investigation [44,45].

From a translational standpoint, the most informative preclinical signals are those linked to disease-relevant endpoints—reduced recurrence, lower metastatic burden, and, in some nanozyme studies, complete tumor regression under clinically plausible dosing conditions [45]. These outcomes argue for cautious early-phase evaluation, particularly in metastasis-prevention and adjuvant settings. These concepts are summarized in Table 3.

## 5. Nanotechnology-Enabled Antioxidant Delivery

Therapeutic redox modulation in BC represents a paradoxical but therapeutically exploitable phenomenon in which redox-active compounds either restore oxidative balance or deliberately perturb it beyond tolerable thresholds to induce cell death. However, a major translational barrier is the poor aqueous solubility, rapid metabolism, low bioavailability, and off-target effects of most antioxidant molecules, including curcumin, resveratrol, and flavonoids. Nanotechnology-based delivery systems have emerged as a powerful strategy to overcome these limitations by enabling controlled pharmacokinetics, tumor-specific accumulation, and microenvironment-responsive release profiles. Recent studies demonstrate that nanomaterials can be engineered to modulate intracellular ROS levels, thereby directly influencing redox signaling pathways, mitochondrial function, and cell fate decisions in BC cells [48]. Importantly, the TME, characterized by elevated ROS, hypoxia, and high glutathione (GSH) levels, provides an ideal context for designing redox-responsive nanotherapeutics that selectively target malignant cells while sparing normal tissues.

### 5.1. Rationale for Nano-Formulations

The rationale for employing nano-formulations in antioxidant-based BC therapy is grounded in both pharmacological and biological considerations. First, many antioxidant molecules exhibit dual redox behavior, acting as ROS scavengers under physiological conditions but inducing oxidative stress at higher concentrations or in cancer-specific environments. This duality necessitates precise spatiotemporal control over drug delivery, which can be achieved through nanocarriers. Second, conventional antioxidant therapy often fails due to insufficient intracellular accumulation and rapid systemic clearance; nanoparticle encapsulation significantly enhances cellular uptake via endocytosis and prolongs circulation time through surface modification (e.g., PEGylation) [49].

From a mechanistic perspective, redox reprogramming is tightly linked to metabolic rewiring in BC, including alterations in mitochondrial respiration, NADPH production, and GSH metabolism. Nano formulations can be tailored to interfere with these pathways—for example, by co-delivering antioxidants with metabolic inhibitors or ferroptosis inducers [50]. Indeed, recent nanotechnology-mediated strategies have demonstrated the ability to disrupt iron homeostasis, lipid peroxidation, and amino acid metabolism, thereby sensitizing tumors to oxidative stress-induced cell death pathways such as ferroptosis [51]. Furthermore, antioxidants such as polyphenols modulate epigenetic regulators (e.g., DNMTs, HDACs), and nano delivery enhances their capacity to reprogram gene expression and tumor-suppressor pathways at therapeutically relevant concentrations [52].

### 5.2. Types of Nanocarriers

A wide spectrum of nanocarriers has been developed for antioxidant delivery in BC, each offering distinct advantages in terms of stability, targeting, and redox modulation. The enhanced permeability and retention effect was characterized in rapidly growing, well-vascularized rodent tumors and does not transfer reliably to older, more stromal and irregularly perfused human tumors; moreover, the assumed transport route is incorrect for most designs, since up to 97% of nanoparticle entry across four murine models and three human tumor types occurred by active trans-endothelial transport rather than through inter-endothelial gaps [53], a saturable process that renders accumulation dose-dependent rather than a simple function of particle size [54]. Extravasation does not ensure distribution: elevated interstitial fluid pressure, dense stroma and irregular perfusion confine nanoparticles to perivascular zones and restrict penetration into hypoxic regions [55], which is a mechanism-specific liability for redox-responsive carriers whose trigger compartments are the least accessible. On contact with plasma or biological fluids, nanoparticles adsorb a biomolecular corona that constitutes their effective biological identity [56,57,58,59] and can abolish ligand recognition, as shown by the loss of target-cell specificity in transferrin-functionalized particles [60,61]; because corona composition varies with individual plasma proteome and disease state, targeting data obtained in protein-free systems have limited predictive value. Off-target deposition remains dominated by the mononuclear phagocyte system, with hepatic clearance, sinusoidal flow deceleration, Kupffer cell capture, and hepatocyte and endothelial uptake as the principal determinants of the dose never available to the tumor [62], and splenic marginal-zone uptake contributing further loss that is largely insensitive to surface modification. Nanoformulation does not abolish immune recognition: anti-PEG antibodies are detectable in treatment-naive populations and are associated with accelerated blood clearance and hypersensitivity [63], while acute infusion reactions to liposomal agents are attributed to complement activation-related pseudoallergy [64]. Carrier fate differs by material class: organic systems degrade to fragments with defined metabolic fate, whereas inorganic redox-active platforms based on cerium, manganese, iron, hafnium, or gadolinium raise questions of persistence, metal release, and chronic tissue retention, requiring dedicated long-term toxicology, with diselenide carriers adding a selenium-specific toxicity profile. Manufacturing imposes a parallel constraint, since performance is defined by critical quality attributes: size distribution, lamellarity, drug loading, physical state of the encapsulated drug and ligand density, whose control was decisive for the first approved liposomal anthracycline [65], to which redox-responsive systems add crosslink density and reductive stability, attributes lacking standardized release specifications and therefore leaving batch variation in trigger sensitivity unquantified. Pharmacokinetically, encapsulation alters the disposition of the carrier rather than the free drug, and total plasma concentration is not equivalent to bioavailable drug, yet discrimination between encapsulated and released payload is rarely reported despite being a regulatory expectation. These constraints also clarify why the three delivery mechanisms should not be conflated. Passive accumulation determines how much material reaches the tumor interstitium; it operates at the vessel wall, is governed by circulation time and perfusion rather than carrier chemistry, and sets the ceiling for everything downstream. Ligand-mediated targeting does not raise that ceiling but acts after extravasation, biasing uptake among already-exposed cells according to antigen density and homogeneity, contingent on corona-dependent preservation of ligand accessibility [57,60,61]. Stimulus-responsive release governs neither accumulation nor uptake but only when and where the payload is liberated, and for redox-triggered systems presupposes a reductive compartment of sufficient and reproducible glutathione concentration, a condition that is spatially heterogeneous, clinically unmeasured, and directionally inverted between platforms, since disulfide-linked prodrug carriers require high tumor glutathione whereas reactive-oxygen-generating systems are antagonized by it. Because these layers are multiplicative rather than additive, effective payload delivery falls well below what any single mechanism-level description implies, and claims should therefore specify the layer to which they apply: demonstrated cleavage under reducing conditions is a property of the linkage, not evidence of tumor selectivity, and receptor binding is a property of the surface, not evidence of accumulation. Consistent with this, ligand-mediated targeting has not translated into clinical benefit in HER2+ BC, where MM-302 showed acceptable tolerability with a 13% overall response rate and 7.4-month median progression-free survival in phase 1 [66] but the randomized phase 2 HERMIONE trial was stopped following a data and safety monitoring board recommendation and futility analysis [67]. Accordingly, nanocarriers are described here as capable of increasing tumor exposure relative to free drug, as having reversed resistance phenotypes preclinically without randomized confirmation, and as redistributing rather than abolishing toxicity, not as ensuring tumor-selective release, overcoming resistance, preventing systemic toxicity, or strictly minimizing off-target injury.

#### 5.2.1. Polymeric Nanoparticles

Polymeric nanoparticles, particularly those based on Poly(lactic-co-glycolic acid) (PLGA) and Polyethylene glycol (PEG), represent a highly advanced and versatile platform for targeted cancer therapy, offering controlled release and improved pharmacokinetic profiles. By incorporating redox-sensitive linkages, these systems can achieve precise, on-demand drug release directly within tumor cells [68,69,70]. PLGA is a biodegradable and biocompatible copolymer that allows for the controlled release of encapsulated drugs over periods ranging from days to weeks. The release rate can be tuned by adjusting the lactic acid-to-glycolic acid ratio, with a 50:50 ratio typically yielding the fastest degradation [71]. The conjugation of PEG (PEGylation) creates a **“corona”** around the nanoparticle, reducing immune system recognition and opsonization. This increases blood circulation time and enhances tumor accumulation through the Enhanced Permeability and Retention (EPR) effect [72,73]. These nanoparticles can be modified with active-targeting ligands, such as antibodies against HER2 (e.g., Herceptin), enabling them to specifically bind to and enter HER2-overexpressing cancer cells via receptor-mediated endocytosis [74]. PEG-PLGA systems can co-encapsulate hydrophobic and hydrophilic drugs, as well as imaging agents, for theranostic applications [75]. Redox-sensitive polymeric Nanoparticle systems incorporate disulfide bonds (–S–S–) into the polymer backbone or crosslinkers [76]. Tumor cells often exhibit a highly reducing environment due to elevated glutathione (GSH) levels, which can be 100 to 1000 times higher than in normal tissue [77]. Upon internalization by tumor cells, the high intracellular GSH concentration triggers a thiol-disulfide exchange reaction, causing rapid cleavage of disulfide bonds, subsequent breakdown of the nanoparticle, and rapid release of the drug payload specifically within the cancer cell [70]. This mechanism reduces premature drug release in the bloodstream, minimizes off-target toxicity, and effectively delivers therapeutic agents to the cytosol, enhancing efficacy against multidrug-resistant cells. Common applications include the delivery of chemotherapeutic agents, such as doxorubicin or paclitaxel, using these smart, responsive nano-platforms [78].

#### 5.2.2. Lipid-Based Nanocarriers

Lipid-based nanocarriers, including liposomes and solid lipid nanoparticles (SLNs), are highly effective, biocompatible platforms for delivering hydrophobic antioxidants, thereby addressing challenges such as low water solubility and poor bioavailability. Their structural similarity to cell membranes allows them to improve cell permeability and increase antioxidant stability [79,80,81]. Engineered liposomes can respond to the unique environment of tumor tissues (often acidic, low-pH, or oxidative) to release their cargo specifically at the site of disease, minimizing damage to healthy tissues [82]. Lipid nanoparticles protect delicate antioxidants from premature degradation, improving their absorption in the gastrointestinal tract and increasing their concentration in blood and tissues [83,84]. Hydrophobic (lipophilic) compounds are encapsulated within the lipid core of SLNs or the phospholipid bilayer of liposomes, enabling easy dispersion in aqueous media [85]. These systems, especially SLNs and nanostructured lipid carriers (NLCs), offer sustained and controlled release profiles [86,87].

#### 5.2.3. Inorganic Nanoparticles

Cerium oxide nanoparticles are a prime example of redox-active inorganic materials, noted for their ability to mimic natural enzymes such as superoxide dismutase (SOD) and catalase [88]. Cerium oxide nanoparticles (nanoceria), for example, exhibit reversible redox cycling between Ce^3+^ and Ce^4+^ states [89], allowing them to function as catalytic antioxidants or pro-oxidants [90] depending on the cellular context. Ce^3+^ is oxidized to Ce^4+^ while scavenging superoxide, acting as an SOD-mimetic [91]. The ability of CeONPs (Cerium oxide nanoparticles) to release oxygen, mediated by Ce^3+^/Ce^4+^ switching, allows them to regulate reactive oxygen and nitrogen species (ROS/RNS) [92]. In BC cells, nanoceria has been shown to induce oxidative stress, mitochondrial dysfunction, and apoptosis, thereby selectively disrupting redox homeostasis [93].

Inorganic nanoparticles, such as cerium oxide (CeO_2_), iron oxide (Fe_2_O_3_), and silica (SiO_2_), possess unique intrinsic redox activity, making them highly valuable in biomedicine and catalysis. These materials can act as artificial enzymes—known as nanozymes—capable of scavenging or generating ROS in a self-regenerating manner, often outperforming small-molecule antioxidants. Iron oxide nanoparticles (IONPs), specifically magnetite (Fe_2_O_3_) and maghemite (γ-Fe_2_O_3_), exhibit intrinsic enzyme-like activity, often termed “nanozymatic” behavior [94,95].

Iron oxide nanozymes can catalyze the decomposition of H_2_O_2_ in the presence of a proton donor, similar to natural peroxidases. They possess dual functionality, exhibiting catalase-like and peroxidase-like activities depending on pH, with activity enhanced by high surface-to-volume ratios [96]. Due to their magnetic properties combined with redox activity, they are used for targeted drug delivery and as anti-inflammatory or anti-tumor agents [97].

While traditionally considered inert, silica nanoparticles (SiO_2_) can be engineered for redox activity, often acting as supports for active species or through surface functionalization [98]. Nonporous silica can be covalently modified with redox-active molecules (e.g., ferrocene or ruthenium complexes) to create heterogeneous catalysts [99]. Silica nanoparticles can exhibit inherent ROS-generating properties that induce oxidative stress in targeted cancer cells [100].

#### 5.2.4. Hybrid and Biomimetic Nanocarriers

Hybrid and biomimetic nanocarriers, such as cell membrane-coated nanoparticles (CMNPs), represent a cutting-edge approach in targeted drug delivery, bridging the gap between synthetic nanoparticle cores (e.g., PLGA, silica, liposomes) and biological functionality. By leveraging cell membrane camouflage—using membranes from red blood cells, platelets, or immune cells—these systems act as “stealth” agents, evading immune surveillance and enhancing tissue-specific targeting [101,102,103,104]. Cell membrane-coated nanoparticles retain surface proteins (such as CD47) from the source cell, which act as “don’t eat me” signals, thereby significantly reducing clearance by the mononuclear phagocyte system (MPS) [101,102]. Membranes from platelets or cancer cells enable homotypic targeting, allowing nanoparticles to accumulate at tumor sites or inflamed areas, thereby increasing local concentration [101,105,106].

Besides actively impairing T-cell cytotoxic function, high levels of ROS furthermore contribute to an immunosuppressive TME by promoting the expansion and functional activity of other immunosuppressive cell populations. High ROS levels favor the transition of macrophages toward an M2-like phenotype [107], characterized by the secretion of anti-inflammatory cytokines, growth factors, and pro-angiogenic mediators. These tumor-associated macrophages (TAMs) support tumor growth. Moreover, ROS-inducing conditions have been shown to upregulate PD-L1 expression in macrophages via NF-κB activation, thereby reinforcing immune suppression [108]. At the same time, ROS further promote the expansion and activity of immunosuppressive cell populations, including regulatory T-cells (T-regs) and MDSCs, thereby facilitating immune escape [107].

### 5.3. ROS-Driven Cellular Senescence

Cellular senescence, referring to the permanent and irreversible arrest of the cell cycle, represents one of the hallmarks of cancer [109]. Senescence can be triggered by various stimuli which influence each other. DNA damage, the shortening of telomers and oxidative stress are known contributors to the development of cellular senescence [110,111]. Physiologically, senescence helps to remodel tissues in developmental phases or after an injury. However, the accumulation of senescent cells can also lead to a pro-inflammatory milieu and to a reduced function of a tissue, which builds an optimal environment for (age-related) diseases including cancer [112].

One key feature of cellular senescence is the formation of the senescence-associated-secretory phenotype (SASP). The SASP comprises a heterogeneous mixture of pro-inflammatory cytokines, chemokines, growth factors and matrix-remodeling factors secreted by senescent cells, which can influence neighboring cells and contribute to chronic inflammation and a tumor-promoting microenvironment [113].

A major driver of cellular senescence and a key component in the maintenance of a senescent state are ROS [114]. On one hand, ROS-induced damages, especially DNA damages lead to the activation of tumor suppressing pathways, for instance p53/p21 and p16 [115] which arrest the cell cycle. ROS can furthermore contribute to SASP-associated components such as pro-inflammatory cytokines through the activation of the NFkB pathway [116].

Cellular senescence has traditionally been recognized as a potent tumor-suppressive mechanism because the stable cell-cycle arrest limits uncontrolled proliferation and aberrant DNA replication. In particular, oncogene-induced senescence (OIS), triggered by persistent activation of oncogenes such as RAS and BRAF, can restrain tumor initiation and progression, while senescent cells may further reinforce this effect through the SASP, cell–cell interactions, and enhanced immune surveillance that facilitate the clearance of senescent tumor cells. Similarly, therapy-induced senescence (TIS) represents an important response to anticancer treatments, including radiotherapy and various chemotherapeutic agents, through which DNA damage and sustained proliferative arrest contribute to tumor growth control. However, senescence is not invariably tumor suppressive. SASP-mediated secretion of inflammatory cytokines, growth factors, and chemokines can promote chronic inflammation and create a microenvironment conducive to tumor progression, whereas age-associated immunosenescence can impair immune surveillance and facilitate malignant transformation. Accordingly, senescence is increasingly viewed as a context-dependent and multifaceted process that can influence tumor initiation, progression, therapeutic resistance, dormancy, and stemness, highlighting its dual role as both a natural barrier to tumorigenesis and a potential contributor to cancer development and treatment resistance [117].

In breast cancer, the context-dependent and opposing effects of cellular senescence are particularly important. While senescence can suppress tumor cell proliferation and limit tumor progression, SASP-mediated signaling from senescent cells may, under specific conditions, promote a pro-tumorigenic microenvironment and contribute to disease progression and therapeutic resistance. Thus, the biological and therapeutic consequences of senescence depend strongly on the cellular and microenvironmental context, emphasizing the importance of selectively exploiting its tumor-suppressive effects while limiting its potential tumor-promoting activities.

### 5.4. Angiogenesis

Besides critically shaping the immune microenvironment in cancer, ROS can contribute to angiogenesis within the TME. Angiogenesis refers to the formation and development of new blood vessels and represents a critical process in (breast) cancer progression, as tumors require a continuous supply of blood, oxygen as well as nutrients to sustain growth and metastasis [118]. ROS are key mediators of angiogenic signaling within the TME and can promote angiogenesis through two pathways: Vascular Endothelial Growth Factor (VEGF)-dependent and VEGF-independent pathways [119].

Under hypoxic conditions, which are common in rapidly growing tumors, ROS levels increase and contribute to the stabilization of hypoxia-inducible factor-1α (HIF-1α) [118]. Stabilized HIF-1α enhances the transcription of pro-angiogenic factors, most notably VEGF [118,120] thereby promoting angiogenesis through enhanced endothelial cell proliferation and migration. VEGF acts on endothelial cells by promoting their proliferation, migration, and organization into new blood vessels [121]. Hydrogen peroxide (H_2_O_2_) upregulates HIF-1α expression in a time- and dose-dependent manner, highlighting the direct contribution of ROS to the amplification of hypoxia-driven angiogenic signaling [120].

Moderate ROS can also activate pro-survival signaling pathways including PI3K/AKT, MAPK, and NF-κB [120,122]. These pathways further support endothelial cell survival, proliferation, and migration, thereby reinforcing angiogenic processes independently of, or in conjunction with, VEGF signaling.

Thus, ROS function as central regulators of angiogenesis in BC by integrating hypoxia signaling with growth factor-mediated and intracellular signaling pathways. Through these mechanisms, ROS contribute to the development of an aberrant tumor vasculature that facilitates tumor progression and metastatic dissemination.

### 5.5. Metabolic Reprogramming

ROS are additionally major mediators of metabolic reprogramming in the TME of BC. In BC cells, moderate ROS levels activate redox-sensitive pathways such as PI3K/AKT/mTOR [123] and promote the stabilization of HIF-1α [118]. Beyond its role in angiogenic signaling, HIF-1α also enhances the expression of glucose transporters and glycolytic enzymes, thereby favoring a glycolytic phenotype even in the presence of oxygen. This allows cancer cells to switch between glycolysis and oxidative metabolism depending on nutrient and oxygen availability [124]. Switching to aerobic glycolysis, also referred to as the Warburg effect, further contributes to meeting the high energy demands of cancer cells [125]. A glycolytic (Warburg-type) phenotype is very common but not universal in primary breast-cancer cells, with the highest frequencies in the more aggressive sub-types (triple-negative and HER2+) [126]. Under hypoxic and oxidative stress conditions, HIF-1α-dependent metabolic rewiring helps to limit excessive mitochondrial ROS production. In this context, the shift toward glycolysis is not only a bioenergetic adaptation but also a protective strategy against excessive ROS, as it reduces mitochondrial superoxide generation while redirecting glucose-derived intermediates into pathways that support antioxidant defense [127]. In BC, persistent glucose uptake and glycolytic activity are closely linked to increased GLUT1 expression and poor prognosis [128]. Therefore, a shift of cancer cells towards glycolysis not only allows meeting the high energy demand of cancer cells [129], but also protects them from ROS-induced cytotoxicity [127].

ROS-mediated metabolic reprogramming not only involves cancer cells but also stromal cells, particularly cancer-associated fibroblasts (CAFs) within the TME [130]. Oxidative stress in CAFs induces a glycolytic phenotype, resembling the metabolic shift observed in cancer cells [131]. As a result, CAFs produce and release energy-rich metabolites such as lactate and pyruvate. Neighboring cancer cells import these metabolites and oxidize them through mitochondrial oxidative phosphorylation, thereby generating ATP for their energy needs. This creates a symbiotic relationship in which stromal cells support tumor cell growth [131]. In BC, high stromal expression of the lactate transporter MCT4 has been associated with this phenotype and with more aggressive disease, particularly in triple-negative tumors [132].

## 6. Overcoming Therapy Resistance

Resistance to therapeutic options remains the main cause for morbidity in BC patients [118], highlighting the urgent need to better understand and overcome therapy resistance in cancer treatment. While many anticancer therapies rely on the induction of oxidative stress to eliminate tumor cells, BC cells frequently develop strategies to restore redox homeostasis and evade cell death. ROS play a dual role in this context, acting both as mediators of therapy-induced cytotoxicity and as drivers of adaptive resistance mechanisms. In response to heightened ROS levels in cancer cells, they also increase their antioxidant response pathways, which diminishes the intended cytotoxicity of increased ROS levels. Cells do not undergo apoptosis and therapy resistance is increased [118].

### 6.1. Chemotherapy Resistance and ROS Adaptation

Chemotherapeutic agents commonly used in BC treatment, such as anthracyclines (e.g., doxorubicin), taxanes (e.g., paclitaxel) or platinum-based drugs (e.g., carboplatin) partly exert their effects by increasing intracellular ROS levels, leading to DNA damage and apoptosis. However, cancer cells can adapt to this oxidative stress by upregulating antioxidant defense systems [118]. A central regulator of this adaptation is the NRF2–KEAP1 signaling pathway, which controls the expression of a wide range of antioxidant and detoxifying enzymes. Activation of NRF2 leads to increased production of glutathione, superoxide dismutase, catalase, and enzymes involved in NADPH generation [133]. This enhanced antioxidant capacity enables cancer cells to neutralize therapy-induced ROS and survive cytotoxic stress. In addition, ROS-mediated activation of survival pathways such as PI3K/AKT and MAPK further promotes resistance by enhancing cell proliferation, inhibiting apoptosis, and facilitating DNA repair [118].

### 6.2. Sensitization Strategies

Given the central role of ROS in therapy resistance, targeting redox homeostasis has emerged as a promising strategy to sensitize BC cells to treatment [134]. One approach focuses on inhibiting antioxidant defense mechanisms in cancer cells. As mentioned, one key regulator of oxidative stress is the NRF2 pathway. Under oxidative stress conditions, NRF2 is stabilized and translocated to the nucleus where the activation of antioxidant pathways is initiated [133]. Tumors often exhibit constantly activated NRF2 trough mutations in the KEAP1/NRF2 pathway, which protects them from oxidative cytotoxicity, and leads to therapeutic resistance [135]. Targeting key regulators such as NRF2 [136] or glutathione metabolism disrupts redox balance and reduces the ability of cancer cells to detoxify ROS. This enhances the efficacy of conventional therapies by preventing adaptive resistance. In one study using hypoxic MCF7 BC cells, Syu et al. demonstrated that ROS-induced activation of NRF2 promotes glutathione-dependent antioxidant responses and contributes to cisplatin resistance, while the pharmacological NRF2 inhibition restored chemosensitivity [137], highlighting the potential to target antioxidant defense mechanisms. Another study by Choi et al. showed that dual inhibition of glutaminase and the cystine/glutamate antiporter xCT enhances the sensitivity of triple-negative BC cells to doxorubicin and cisplatin by depleting glutathione and increasing oxidative stress, both in vitro and in an in vivo mouse model [138].

In addition, nanotechnology-based strategies offer new opportunities for precise modulation of ROS within tumors. Redox-responsive nanocarriers can deliver therapeutic agents specifically to the tumor site, enabling controlled ROS generation or antioxidant inhibition while minimizing systemic toxicity [107]. For example, Zhang et al. engineered a redox-responsive nanocarrier that repolarizes immunosuppressive TAMs to M1-like phenotype while simultaneously scavenging excess ROS and silencing PD-L1, thereby enhancing systemic antitumor immunity in mouse models [139].

## 7. Preclinical and Clinical Evidence

Nanotechnology-enabled redox modulation strategies in BC have progressed substantially from mechanistic in vitro studies to complex in vivo models and early-phase clinical evaluations. These approaches aim to exploit oxidative vulnerabilities in tumor cells while minimizing systemic toxicity, thereby bridging the gap between experimental redox biology and translational oncology. Clinical experience with redox-based nanomaterials in BC is, at present, limited to three distinct and much narrower settings, none of which provides evidence for redox-responsive drug delivery [140]. First, antioxidant nanocarriers have entered early-phase evaluation in a supportive-care rather than antitumor indication: a randomized, double-blind, split-body, placebo-controlled phase Ib study of APN201, a liposomal formulation of recombinant human Cu/Zn superoxide dismutase, was conducted in 20 women receiving adjuvant whole-breast radiotherapy after breast-conserving surgery, and reported acceptable tolerability with preliminary signals on erythema and time to grade 2 dermatitis. Second, ROS-amplifying inorganic nanoparticles have been included in histologically mixed cohorts of BC patients. The gadolinium-based nanoparticle AGuIX was evaluated with whole-brain radiotherapy in patients with multiple brain metastases; breast primaries represented 2 of 15 patients in the phase I NANO-RAD study and 14% of the randomized phase II NANORAD2 cohort. NANORAD2 did not meet its primary endpoint, with best intracranial response rates of 58% versus 49% (*p* = 0.42) and no overall survival benefit. This negative result is explicitly reported here, since selective citation of positive early-phase data would misrepresent the current evidence base [141]. Third, redox-based radiosensitization itself, independent of nanoformulation, has reached randomized phase II in BC through intratumoral hydrogen peroxide (KORTUC, NCT03946202), which is a hydrogel rather than a nanocarrier and therefore provides mechanistic, not formulation-level, support [141,142].

Therapeutic redox modulation in BC has a context-dependent role: in some models, antioxidants suppress ROS-driven proliferation, DNA damage, inflammation, EMT, and therapy-related toxicity; in others, excessive antioxidant supplementation may protect tumor cells from oxidative stress and support survival, metastasis, or treatment resistance. Therefore, the therapeutic value of antioxidants depends on tumor subtype, redox status, dose, timing, delivery system, and combination with chemotherapy, radiotherapy, immunotherapy, or nanomedicine. Recent reviews emphasize that BC cells maintain a delicate ROS threshold: moderate ROS supports signaling and invasion, whereas excessive ROS induces apoptosis, ferroptosis, or other regulated cell death pathways.

### 7.1. In Vitro and In Vivo Models

Preclinical investigations have demonstrated that redox-responsive nanomedicines effectively modulate intracellular oxidative stress in BC models. In vitro studies using cell lines such as MCF-7 (estrogen-receptor positive) and MDA-MB-231 (triple-negative) have shown that nanoparticle-mediated delivery of redox-active compounds can induce ROS accumulation, mitochondrial dysfunction, and subsequent activation of ferroptosis or apoptosis pathways. For example, iron-based nanomaterials and Fenton reaction catalysts have been widely used to selectively increase ROS levels and trigger lipid peroxidation in cancer cells [143,144].

In vitro studies commonly use BC cell lines such as Michigan Cancer Foundation-7 (MCF-7), T47D, SK-BR-3, MDA-MB-231, BT-549, 4T1, and MCF-10A to evaluate the effects of antioxidants on ROS levels, mitochondrial membrane potential, glutathione metabolism, apoptosis, ferroptosis, autophagy, migration, invasion, and drug resistance. TNBC models are particularly relevant because they often show elevated mitochondrial ROS and stronger dependence on redox signaling. In such systems, antioxidants such as N-acetylcysteine, vitamin C derivatives, polyphenols, flavonoids, curcumin, resveratrol, melatonin, selenium compounds, and antioxidant enzyme mimetics may either inhibit tumor-promoting oxidative signaling or interfere with ROS-dependent cytotoxic therapies [145].

Therapeutic redox modulation in BC involves manipulating the cellular redox balance—specifically, increasing antioxidant defense mechanisms (such as NRF2 activation) or using pro-oxidant strategies to disrupt the high redox threshold of cancer cells. In vivo models are critical for evaluating these strategies, with a focus on tumor growth, metastatic progression, and modulation of the TME. In vivo models include xenograft models, orthotopic mammary tumor models, 4T1 syngeneic metastatic models, and genetically engineered mouse models [146,147]. These models are used to assess tumor growth, lung metastasis, angiogenesis, immune infiltration, oxidative damage markers, and systemic toxicity [148]. Importantly, recent work has reported that systemic N-acetylcysteine (NAC) administration can enhance pulmonary metastasis in murine BC models, showing that antioxidant therapy may become risky when it lowers oxidative barriers that normally restrict metastatic colonization [149]. In vivo studies typically focus on the following to determine the effect of redox reprogramming:

(1) Tumor growth and metastasis: Monitoring tumor volume (using calipers or imaging) and assessing the number/size of metastatic lesions, particularly in the lungs [150,151].
(2) Angiogenesis: Evaluating the development of new blood vessels in the tumor, often regulated by redox-sensitive factors like HIF-1α [9,152,153].
(3) Immune infiltration: Assessing changes in the immune landscape (e.g., macrophage polarization to M1/M2) following redox-based therapy, such as using nanoparticle-based antioxidants [93,154,155].
(4) Oxidative damage markers: Measuring markers of oxidative stress, such as malondialdehyde (MDA) or 4-hydroxy-2-nonenal (4HNE), to confirm that the intervention successfully increased ROS in cancer cells [156].
(5) Systemic toxicity: Assessing the impact of treatments on the overall health of the mice, including weight change and liver/kidney damage [154,156].

The basic redox targets in in vivo studies are:

(1) NRF2 Pathway: Studies demonstrate that inhibiting NRF2 can sensitize tumors to therapy by lowering the antioxidant threshold.
(2) Mitochondrial oxidative phosphorylation (OXPHOS): Targeting mitochondrial function with antioxidants (e.g., MitoQ) can inhibit metastasis, particularly in TNBC.
(3) Cancer-associated adipose tissue (CAAT) plays a critical role in the tumor microenvironment. When healthy adipocytes interact with cancer cells, they undergo a profound “dedifferentiation” process, transforming into hyper-catabolic cancer-associated adipocytes [157,158,159].

### 7.2. Clinical Potential and Safety

Clinically, antioxidants have been investigated for three major purposes: (1) cancer prevention, (2) adjuvant support to reduce therapy-induced oxidative toxicity, and (3) direct redox-based anticancer therapy. Some clinical observations suggest potential benefits of selected antioxidants in reducing treatment-related side effects or modulating tumor metabolism; for example, a pilot BC study reported that NAC reduced carcinoma cell proliferation markers in early-stage patients [160]. However, clinical translation remains controversial. Antioxidant supplementation during chemotherapy or radiotherapy may reduce oxidative damage in normal tissues, but it may also reduce ROS-mediated tumor killing. Therefore, non-selective, high-dose, long-term antioxidant use should not be assumed safe in BC patients. Current evidence supports a more cautious approach: antioxidant-based therapy should be personalized according to tumor subtype, baseline oxidative stress, treatment regimen, metastatic risk, and biomarker profile [161]. Overall, Therapeutic redox modulation is promising, but it should be developed as a precision redox therapy rather than generalized supplementation. The safest and most rational future direction is targeted antioxidant or redox-modulating nanomedicine, supported by subtype-specific biomarkers such as ROS level, GSH/GSSG ratio, NRF2/KEAP1 activity, GPX4 status, mitochondrial dysfunction, ferroptosis sensitivity, and immune-redox signatures. Although redox indicators such as ROS accumulation, GSH/GSSG balance, NRF2 activation, and GPX4 status are valuable for understanding ferroptosis sensitivity in laboratory settings, none of these markers are currently validated or approved for routine clinical use in selecting targeted therapies for breast cancer patients [162,163]. These remain exploratory preclinical targets that require standardized analytical measurements and clinical threshold validation in future clinical trials.

Despite promising preclinical results, the clinical translation of redox-modulating nanomedicines for BC remains in its early stages. Several nanoparticle-based drug delivery systems, including liposomal formulations and albumin-bound nanoparticles, have already been approved for clinical use, demonstrating the feasibility of nanomedicine in oncology. A representative example is Doxil^®^, a PEGylated liposomal formulation of doxorubicin that was developed to improve the pharmacokinetic and safety profile of doxorubicin, including reducing anthracycline-associated cardiotoxicity compared with conventional doxorubicin. However, the established clinical use of such nanomedicines should be clearly distinguished from redox-responsive and redox-modulating nanoplatforms, which remain predominantly investigational and have not yet achieved comparable clinical or regulatory validation [164]. Doxil^®^ was reported to substantially inhibit tumor growth and prolong survival in preclinical models [165]. Moreover, compared with conventional free doxorubicin, Doxil was associated with an approximately threefold reduction in the risk of cardiotoxicity [166]. In a Phase III clinical trial involving 22,509 patients with metastatic breast cancer, Doxil^®^, administered at 50 mg/m^2^ demonstrated comparable progression-free and overall survival outcomes to conventional doxorubicin administered at 60 mg/m^2^, while exhibiting a significantly lower incidence of cardiotoxicity [166]. By reducing cardiotoxicity, Doxil^®^, may alleviate the limitations associated with cumulative doxorubicin dosing, thereby enabling prolonged treatment at a lower risk of cardiotoxicity and substantially expanding the therapeutic versatility of this drug. Doxil^®^, has been investigated in combination with several chemotherapeutic agents, including cyclophosphamide and 5-fluorouracil, cisplatin and infusional fluorouracil, and cyclophosphamide followed by paclitaxel, as well as with targeted therapies such as trastuzumab for the treatment of advanced breast cancer in clinical trials [167,168,169,170]. These studies demonstrated favorable efficacy and a relatively low toxicity profile, including with respect to cardiotoxicity, even in elderly patients and those at increased risk of cardiotoxicity [171]. It should be noted that a recent meta-analysis showed that, although Doxil^®^, substantially reduces cardiotoxicity, it may also introduce treatment-related adverse effects, such as cutaneous toxicity and mucositis; however, these adverse effects are clinically less serious than cardiotoxicity [171]. Overall, these findings provide supportive clinical evidence that nanomedicine-based formulations can be effectively and safely integrated with standard anticancer drug therapies [172]. Furthermore, Abraxane^®^—an albumin-bound nanoparticle formulation of paclitaxel—facilitates drug delivery and has demonstrated enhanced efficacy in metastatic breast cancer compared to conventional solvent-based paclitaxel [173]. Abraxane, also referred to as nanoparticle albumin-bound paclitaxel or nab-paclitaxel, is a nanoparticle-based formulation of paclitaxel [174]. In this formulation, paclitaxel is noncovalently associated with approximately 130 nm nanoparticles prepared from human serum albumin. Abraxane^®^ was initially developed as a solvent-free formulation of paclitaxel to overcome the limitations associated with Cremophor EL and ethanol, which are used to solubilize conventional paclitaxel and are implicated in a high incidence of hypersensitivity reactions requiring premedication [175]. Importantly, Abraxane^®^ not only eliminates solvent- and surfactant-associated adverse effects and obviates the need for routine premedication, but also exploits the physiological interactions of albumin with the gp60/caveolin-1 receptor pathway to facilitate rapid and preferential delivery and accumulation of paclitaxel within tumor tissue [175,176,177]. Compared with solvent-based paclitaxel, Abraxane^®^ has been reported to achieve a 9-fold greater tissue penetration of paclitaxel through transporter-mediated pathways, a 33% increase in intratumoral drug concentration, a 10-fold higher mean maximum concentration of free paclitaxel, and a 4-fold lower elimination rate [176,177,178]. These pharmacokinetic and delivery advantages have also translated into favorable clinical outcomes. In the GeparSepto trial, [179] which included 1229 women with previously untreated unilateral or bilateral primary invasive breast cancer, replacement of solvent-based paclitaxel with nab-paclitaxel significantly increased the proportion of patients achieving a pathologic complete response following anthracycline-based chemotherapy. These findings further supported the potential use of nab-paclitaxel as an alternative to solvent-based paclitaxel in the primary treatment of breast cancer. The combination of Abraxane^®^ with established anticancer agents has also been investigated in different breast cancer settings. In patients with HER2-positive breast cancer, treatment with Abraxane^®^ in combination with carboplatin and trastuzumab demonstrated favorable efficacy and an overall acceptable safety profile [180]. Similarly, another clinical trial showed that the addition of bevacizumab to Abraxane^®^ before dose-dense doxorubicin/cyclophosphamide significantly increased the pathologic complete response rate compared with chemotherapy alone in patients with triple-negative, locally advanced, or inflammatory breast cancer [181]. Overall, Abraxane^®^ has demonstrated comparable or improved efficacy relative to solvent-based paclitaxel in breast cancer treatment and, similar to Doxil^®^, can be incorporated into combination regimens with conventional chemotherapeutic and targeted agents.

Emerging clinical strategies are exploring redox-specific targeting. Nanocarriers designed to respond to tumor-specific oxidative conditions are under investigation for their ability to selectively release therapeutic agents while minimizing off-target effects. However, challenges remain, including nanoparticle biodistribution, long-term toxicity, immunogenicity, and variability in patient-specific tumor microenvironments [182]. Safety considerations are particularly critical, as excessive ROS generation can damage healthy tissues and induce systemic oxidative stress. Therefore, precise control over redox modulation is essential. Advances in biomimetic nanocarriers and biodegradable materials are addressing these concerns by improving biocompatibility and clearance profiles [182,183].

Biomarker-guided personalized redox nanomedicine is in an early translational stage, underscoring the need for ongoing efforts to bridge preclinical findings and clinical application. Progress from concept to practice will depend on structured criteria in four domains: patient stratification, analytically validated quantitative redox biochemistry, companion diagnostic co-development, and prospective clinical trial validation [140,184,185,186]. Overall, while nanotechnology-enabled redox therapies hold significant promise, their successful clinical translation will depend on developing personalized strategies that account for tumor redox heterogeneity and on rigorous evaluation of safety and efficacy in large-scale clinical trials.

Although it is known that various studies on cancer research are still ongoing today [187,188,189,190,191,192], to date, no redox-responsive therapeutic delivery strategy has received regulatory approval from the FDA (Food and Drug Administration) or EMA (European Medicines Agency), nor has definitive clinical efficacy been established in human trials for breast cancer. Although emerging approaches designed to exploit intracellular redox gradients, including elevated glutathione and hydrogen peroxide levels, have demonstrated substantial therapeutic potential in preclinical mouse models, their clinical translation remains at the investigational stage [193,194].

## 8. Challenges and Controversies

Redox-based interventions in oncology are constrained by two linked problems: the antioxidant paradox and the strong dependence of outcome on dose and timing relative to tumor biology and treatment.

### 8.1. The Antioxidant Paradox

The antioxidant paradox refers to the observation that antioxidant systems can be protective in some contexts yet pro-tumoral in others. In established tumors, relieving oxidative stress may preserve survival, proliferation, and metastatic competence rather than suppress them [16]. Sayin et al. [195] were among the first to show clearly that supplementation with common antioxidants, including N-acetylcysteine and vitamin E, could accelerate tumor progression in mouse models by blunting ROS-dependent p53 activation and DNA-damage signaling. This foundational study used KRAS- and B-Raf-driven mouse models of lung cancer, not breast cancer; the mechanism it defined—antioxidant blunting of ROS-dependent p53 signaling—is biologically plausible in p53-competent breast tumors but has not been directly demonstrated in breast cancer models, and the extrapolation below should be read as a working hypothesis motivated by the lung cancer data rather than established breast-cancer evidence. That study prompted a broader reassessment of antioxidant exposure in established cancers, including BC.

BC models reveal the paradox in two main ways. First, antioxidant supplementation or thiol boosting can, in some contexts, promote dissemination. Studies in non- BCs (melanoma) showed that systemic NAC increased metastasis in mice by increasing cellular GSH and enabling survival during dissemination [196]. The results of these studies have motivated BC researchers to test analogous interventions. Second, selective loss of intracellular antioxidant enzymes can also promote malignancy. Ren et al. [16] showed in robust mammary tumor models that loss of glutathione peroxidase 2 (GPx2) paradoxically increased intratumoral ROS, stabilised HIF-1α, disrupted angiogenesis, and promoted metastasis. Thus, both increasing and decreasing antioxidant capacity can promote tumor progression, depending on which redox node is perturbed and when the perturbation occurs. Crucially, breast-cancer-relevant work has also identified mechanistic determinants that explain divergent outcomes: (i) which redox node is modulated (e.g., mitochondrial ROS vs. cytosolic GSH), (ii) the cellular compartment targeted, and (iii) interactions with tumor suppressors and stress sensors (e.g., p53, NRF2, HIF-1α). For instance, targeted mitochondrial antioxidants (MitoQ) were shown to prevent metastatic take and recurrence in aggressive BC mouse models, indicating that compartment-specific antioxidant strategies can be beneficial while systemic thiol donors may be harmful [14,47]. The paradox is therefore not arbitrary. It depends on the redox node being perturbed, its subcellular location, and the timing of intervention relative to tumor evolution and treatment.

### 8.2. Dose and Timing Issues

Dose and timing have long been recognized as important determinants of antioxidant activity in cancer, but recent clinical and experimental data define these principles more clearly. Clinical reports from a large prospective ancillary analysis of the SWOG S0221 trials, which included approximately 1134 patients, highlighted that the use of antioxidant supplements both before and during adjuvant chemotherapy was associated with an increased hazard of recurrence and death, with an adjusted hazard ratio of approximately 1.4 for recurrence [197]. This patient-level dataset represents one of the most compelling clinical demonstrations that the timing of antioxidant administration concurrent with therapy versus post-treatment can materially alter clinical outcomes in early BC.

Preclinical studies conducted over the last five to seven years have provided mechanistic explanations for the observations reported in the SWOG analysis. Controlled animal and cell-based experiments have shown that antioxidants can protect tumor cells from therapy-induced oxidative death when administered during cytotoxic treatment. Qin et al. showed that NRF2 activation protects TNBC stem-like cells from ionizing radiation, and that inhibition of NRF2 sensitizes these cells to radiation by increasing intracellular ROS and impairing DNA repair pathways [198]. These observations help explain why antioxidant activity or NRF2 upregulation during radiotherapy or chemotherapy can be detrimental, as suppression of therapy-induced ROS can attenuate the oxidative signaling required for effective tumor cytotoxicity.

Dose-dependent biphasic responses are a consistent feature of redox-active compounds. Nitroxides such as Tempol exhibit antioxidant effects at low concentrations, promoting cell survival, while higher concentrations induce pro-oxidant activity, apoptosis, and metabolic collapse [40]. This biphasic behavior illustrates that the same chemical entity may exert protective or cytotoxic effects depending on dose, reinforcing the importance of pharmacodynamic characterization in preclinical models.

Compartmental targeting further modifies biological outcome. Mitochondria-targeted antioxidants such as MitoQ have been shown to reduce metastatic seeding and recurrence in aggressive BC models without impairing standard chemotherapy efficacy in vitro, indicating that modulation of mitochondrial ROS can suppress dissemination without interfering with cytotoxic ROS generated during treatment [14]. In contrast, broad thiol donors such as N-acetylcysteine have been associated with increased metastatic behavior in other tumor models [196], and clinical observational data indicate potential harm when antioxidants are administered concurrently with chemotherapy [197]. These data indicate that therapeutic impact depends strongly on the subcellular site of ROS modulation. Timing relative to microenvironmental exposures also influences outcomes. Transient hypoxia imprints ROS-resistant metastatic competence, with antioxidant interventions exerting divergent effects depending on their timing relative to hypoxic episodes [18]. The same antioxidant may therefore suppress or facilitate metastasis depending on its temporal relationship to microenvironmental stress.

Finally, high-dose pro-oxidant strategies behave differently from classical antioxidant supplementation. Pharmacological ascorbate administered intravenously generates extracellular hydrogen peroxide within tumor interstitia and has been shown in preclinical models to enhance chemosensitivity and produce selective tumor cytotoxicity [199]. This distinction emphasizes that dose and route of administration—oral low-dose versus intravenous pharmacologic dosing—can invert the mechanism of action from antioxidant to pro-oxidant.

**Figure 3:**
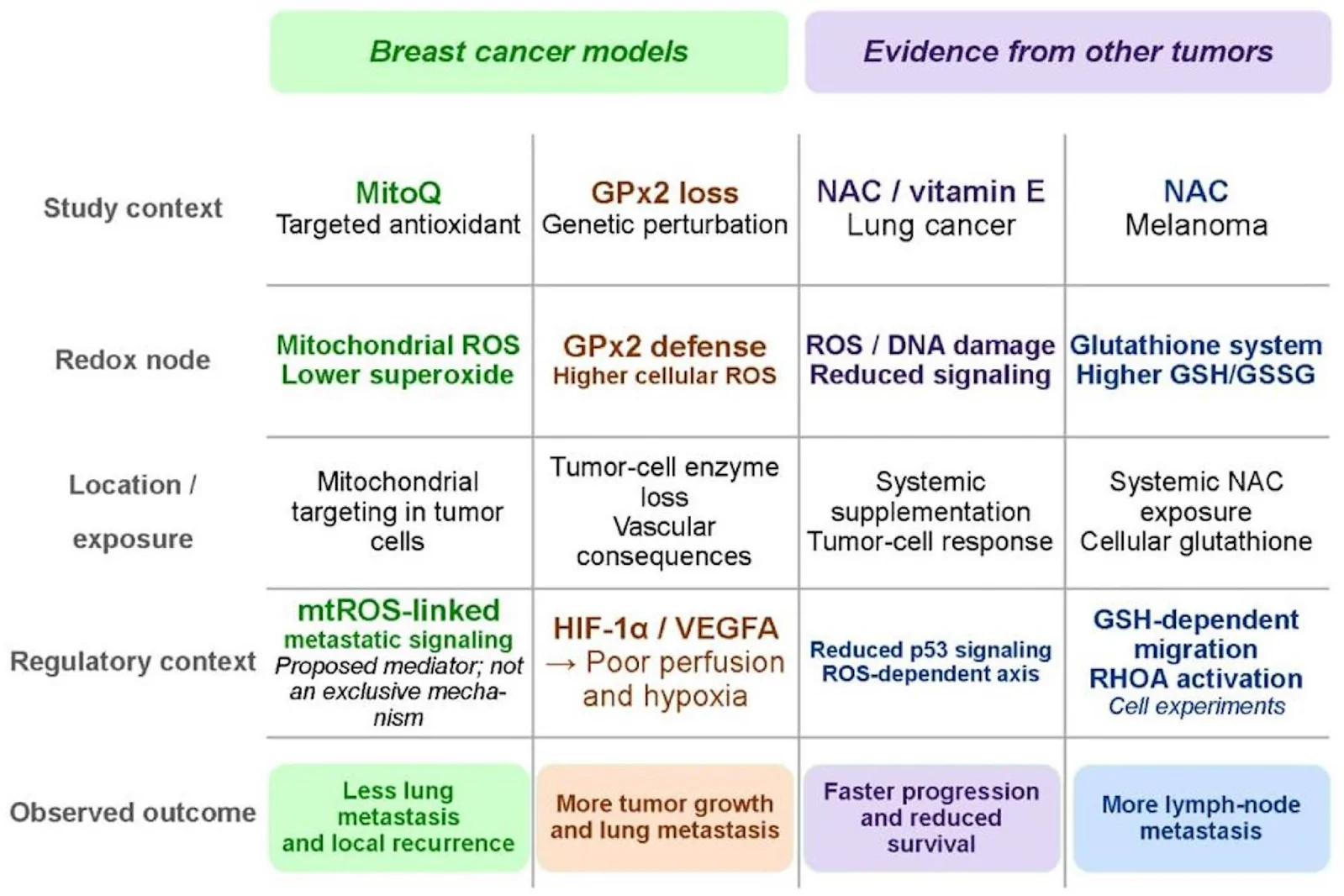
The antioxidant paradox in cancer: context-dependent effects of ROS modulation. Matrix comparing four interventions that perturb redox balance in opposite directions, showing that outcome depends on which redox node is perturbed, in which compartment and in which model, rather than on the direction of ROS change. Symbol and colour key: the header bands separate studies in breast cancer models (green, left) from studies in other tumor types (purple, right), a distinction essential to interpretation since the two groups do not carry equal evidential weight for breast cancer; columns are study contexts and rows the dimensions of comparison, with each column colour-coded consistently across rows; the arrow (→) denotes a causal sequence reported in that study rather than a correlation; and italicized text marks qualifications added in this review, not claims of the original studies. Demonstrated in breast cancer: MitoQ reduced mitochondrial superoxide, lung metastasis and local recurrence in TNBC and MMTV-PyMT models; GPx2 loss raised ROS, stabilized HIF-1α, caused VEGFA-dependent vascular abnormalization and increased tumor growth and metastasis. Proposed rather than demonstrated: the two right-hand columns derive from lung cancer and melanoma models and serve as mechanistic precedent only; neither axis has been tested in breast tumors. Columns differ in species, dose, route and timing and are not quantitatively comparable.

Several practical implications emerge. Routine systemic antioxidant supplementation during cytotoxic therapy should be approached with caution. Compartment-targeted strategies may prove more useful, but only if dosing and timing are matched carefully to treatment context and microenvironmental stress history. Dosing strategies must account explicitly for biphasic dose-response relationships and the timing of microenvironmental stress exposures to avoid unintended promotion of tumor survival or dissemination. The accumulated evidence illustrates the context-dependent nature of antioxidant interventions in cancer and provides a mechanistic framework for the so-called antioxidant paradox. Context-dependent effects of antioxidant interventions in BC are shown in Table 4. Context-dependent effects of ROS modulation are shown in Figure 3.

## 9. Emerging Horizons

Antioxidant-based redox nanomedical systems have emerged as a promising approach in BC therapy by modulating intratumoral oxidative stress while minimizing damage to healthy tissues. As a potential component of personalized therapeutic strategies, these systems may help overcome drug resistance and improve therapeutic efficacy. In BC treatment, antioxidant nanocarriers can potentially counteract chemotherapy resistance by restoring redox homeostasis and mitigating the excessive oxidative stress associated with treatment-induced cellular damage. The literature reports that antioxidant-based redox nanomedicine systems offer a promising approach for breast cancer treatment by modulating intratumoral oxidative stress while minimizing damage to healthy tissues. Furthermore, antioxidant nanocarriers used in breast cancer treatment may potentially overcome chemotherapy resistance by restoring redox homeostasis and alleviating the excessive oxidative stress associated with treatment-induced cellular damage [200].

Key future visions of antioxidant-induced redox nanomedical systems can be summarized as follows:

**Personalized Treatment and Sensitivity:** Nanotechnology offers “smart” drug delivery systems customized to the patient’s tumor structure and sensitive to the redox status of the tumor cell.

**Overcoming Drug Resistance:** Combining antioxidant nano-systems with chemotherapy agents may help overcome treatment resistance that develops in cancer cells.

**Reducing Side Effects:** Nanoparticle-based drug delivery systems offer a promising strategy to mitigate the systemic toxicities, such as cardiotoxicity, typically associated with conventional chemotherapeutics. By modulating the pharmacokinetic profiles and biodistribution of encapsulated agents, these platforms can preferentially enhance tumor accumulation through passive or active targeting mechanisms, thereby potentially reducing off-target exposure in healthy tissues.

**Therapeutic and Diagnostic (Theranostic) Approach:** Theranostic nanoplatforms integrate diagnostic and therapeutic modalities into a single system, offering the potential for simultaneous tumor imaging and controlled drug delivery. By modulating biodistribution, these engineered structures can enhance target site localization while minimizing systemic exposure.

**Destruction of Tumor Stem Cells:** The challenges encountered in the treatment of cancer stem cells can be overcome by using redox-sensitive nano-prodrugs to either differentiate or directly destroy these cells [201,202].

Especially, personalized redox nanomedicine represents a transformative paradigm in targeted therapy. It leverages nanomaterials to sense and respond to the unique oxidative or reductive microenvironments of individual patients. This approach has the potential to improve therapeutic efficacy—especially in oncology—while reducing off-target toxicity and damage to healthy tissues [186,203,204]. Personalized redox nanomedicine targets chemical differences (e.g., high oxidative stress) in the microenvironment of tumor cells in BC treatment, with the potential to promote preferential drug release within tumor tissues. This approach potentially mitigates systemic toxicity and circumvents established resistance mechanisms. Ultimately, the integration of artificial intelligence with multifunctional, stimuli-responsive nanocarriers aims to offer a more precise framework for optimizing future oncological therapeutic strategies. Among the key visions for the future of personalized redox nanomedicine are:

• **Intracellular Targeting (Oxidative Stress):** BC cells contain significantly higher levels of ROS and antioxidant molecules compared to normal cells. Future nanosystems will utilize this difference to deliver drugs via redox reactions.
• **Personalized Treatment and Diagnosis (Theranostics):** Nanotechnology is designed specifically for the patient’s tumor profile. This allows a single system to both target the tumor and deliver the drug, as well as monitor the response to treatment in real time.
• **Intelligent Designs and Multi-Omics Integration:** Biocompatible nanoparticles developed through the integration of intelligent design and multi-omics data may help address mechanisms of drug resistance, potentially increasing the likelihood of therapeutic success, especially in aggressive subtypes such as TNBC [205,206,207,208].

## 10. Conclusions

BC redox biology is far more complex than a simple imbalance between oxidants and antioxidants. Reactive oxygen species function as dynamic signaling mediators that shape tumor progression, metastatic dissemination, immune escape, and therapeutic resistance, making redox homeostasis a context-dependent determinant of therapeutic response. Accordingly, antioxidants should not be viewed exclusively as cytoprotective scavengers but as potential modulators of tumor redox states. This perspective is particularly relevant to BC, where ER+, HER2+, and triple-negative tumors exhibit distinct redox dependencies and stress-adaptation mechanisms. Such heterogeneity helps explain the inconsistent effects of indiscriminate antioxidant interventions and supports the development of selective, subtype-informed therapeutic redox modulation strategies.

Nanotechnology-based delivery systems provide an important translational opportunity by enabling more controlled spatial and temporal regulation of redox-active agents. Redox-responsive nanocarriers, nanozymes, and biomimetic platforms can improve drug stability and tumor exposure while potentially reducing systemic toxicity and enhancing therapeutic selectivity. However, their clinical translation remains constrained by limited control over intratumoral distribution, biological barriers, variable tumor microenvironments, nano–bio interactions, and uncertainties regarding the long-term safety and pharmacokinetics of nanomaterials. Nanozymes in particular require further optimization of catalytic activity, substrate specificity, stability, and behavior within heterogeneous tumor microenvironments. Similarly, efficient delivery remains dependent on overcoming biological barriers that can limit tumor penetration and intracellular access. These challenges highlight the need to move beyond proof-of-concept efficacy toward rigorous evaluation of pharmacological behavior, reproducibility, biodistribution, toxicity, and clinically relevant models.

Future clinical development should therefore integrate molecular redox profiling with biomarker-guided patient stratification to identify tumors with actionable oxidative vulnerabilities. Real-time assessment of tumor redox dynamics, together with subtype-specific nanomedicine design, may further enable treatment selection and adaptive therapeutic strategies. Overall, therapeutic redox modulation of tumors offers a promising framework for precision BC therapy, but its clinical impact will depend on translating mechanistic redox insights into safe, biomarker-driven, and clinically feasible delivery platforms. Rather than broadly suppressing oxidative stress, future approaches should selectively exploit tumor-specific redox vulnerabilities while preserving normal tissue homeostasis, with the ultimate goal of improving therapeutic response and overcoming resistance and recurrence.

## Figures and Tables

**Table 1:** Evolution of redox homeostasis concepts in BC biology.

Aspect	Earlier Framing (General Redox Biology)	Breast-Cancer-Specific Evidence (2019–2024)	References
**Mitochondrial ROS (mtROS)**	Mostly viewed as metabolic by-products of oxidative phosphorylation	Demonstrated to actively regulate metastatic fitness and colonization; modulation of mitochondrial dynamics (e.g., DRP1 inhibition or mitochondrial fusion) significantly alters metastasis in vivo	[12,13,14]
**ROS compartmentalization**	ROS sources often considered interchangeable contributors to global oxidative stress	Evidence indicates that mitochondrial, NOX-derived, and microenvironment-derived ROS regulate distinct signaling networks and metastatic processes	[15,16]
**Hypoxia–ROS interactions**	Hypoxia primarily linked to HIF activation and tumor aggressiveness	Fate-mapping studies reveal a persistent “hypoxic memory” generating ROS-resistant tumor cell populations with increased metastatic potential	[15,26]
**NADPH oxidases (NOX)**	Recognized as ROS-generating enzymes in cancer cells	BC studies show NOX4-mediated ROS promotes PTEN oxidation, PI3K/AKT activation, and stromal remodeling that facilitate invasion and angiogenesis	[15,16]
**Subtype-specific redox biology**	Limited understanding of ROS differences between BC subtypes	TNBC shows strong dependence on mitochondrial redox plasticity, whereas ER+ and HER2+ tumors display distinct metabolic-redox vulnerabilities	[12,13,14,23]
**Therapeutic implications**	ROS modulation primarily approached through systemic antioxidant strategies	Emerging approaches emphasize compartment-targeted redox interventions, including mitochondrial antioxidants, modulation of mitochondrial dynamics, and ferroptosis-based therapies	[14,23]

**Table 2:** Redox-regulated signaling pathways in BC.

Signaling Pathway	Classical Understanding	Recent Insights (2019–2024)	Biological Consequence	References
**NRF2-KEAP1 axis**	Master regulator of antioxidant responses protecting cells from oxidative damage	NRF2 maintains therapy-resistant cancer stem-like cells and promotes radioresistance in TNBC; pharmacologic inhibition sensitizes tumors to ROS-mediated therapy	Redox buffering and therapy resistance	[27]
**p53 redox regulation**	p53 coordinates oxidative stress responses and apoptosis	Mutant p53 actively rewires metabolic and antioxidant networks, increasing redox tolerance and metastatic competence	Metabolic plasticity and ROS tolerance	[17,18]
**Ferroptosis regulation**	Ferroptosis associated with iron-dependent lipid peroxidation	p53 status determines ferroptosis susceptibility through regulation of SLC7A11 and glutathione metabolism	Context-dependent ferroptotic vulnerability	[17,23]
**PI3K/AKT signaling**	ROS can oxidize PTEN and activate PI3K/AKT signaling	Mitochondrial ROS regulate AKT/ERK signaling through mitochondrial dynamics and redox-dependent phosphatase inactivation	Sustained oncogenic signaling	[13,34]
**Metabolic-redox feedback**	Signaling and metabolism considered loosely connected	AKT signaling increases NADPH production and NRF2 activation, reinforcing antioxidant capacity	Stabilization of redox-adapted tumor state	[16,34]
**Hypoxia signaling (HIF-1α)**	HIF-1α stabilized primarily by oxygen deprivation	ROS imbalance can stabilize HIF-1α independently of oxygen levels, linking oxidative stress to pseudo-hypoxic signaling	Metastatic adaptation and angiogenesis	[15,16,18]

**Table 3:** Redox-targeting therapeutic strategies in BC.

Strategy	Representative Agents/Examples	Mechanism of Redox Modulation	Key Findings (Recent Studies)	Translational Implication	Evidence Level	References
**Natural antioxidants**	Resveratrol, curcumin, quercetin	Modulation of intracellular ROS and antioxidant signaling	Nanoformulated resveratrol and phytochemical combinations suppress tumor growth and modulate tumor immune microenvironment in TNBC models	Potential adjuncts to therapy when optimized for bioavailability	Preclinical only—in vitro + in vivo TNBC xenograft models	[46]
**Nanoformulated phytochemicals**	Liposomal resveratrol, nanocarrier curcumin	Improved delivery and tumor accumulation of redox-active compounds	Nanoformulations significantly enhance antitumor efficacy in vivo compared with free compounds	Overcomes pharmacokinetic limitations of natural antioxidants	Preclinical only—in vivo efficacy studies	[46]
**Mitochondria-targeted antioxidants**	MitoQ, Mn-based antioxidants	Selective modulation of mitochondrial ROS (mtROS)	Reduction in metastatic colonization and recurrence in preclinical TNBC models	Potential strategy for metastasis prevention	Preclinical only—in vivo TNBC/metastasis models	[40,47]
**Synthetic small-molecule antioxidants**	Tempol and related nitroxides	Redox cycling and modulation of cellular metabolism	Dose-dependent biphasic effects: antioxidant at low doses, pro-oxidant and cytotoxic at higher doses	Requires careful dose optimization	Preclinical only—in vitro/in vivo dose–response studies	[40]
**Nanozyme-based therapies**	CeO_2_ nanozymes, Pt-based nanozymes, Ru nanozymes	Catalytic ROS generation or oxygen modulation in tumor microenvironment	Catalytic nanozymes induce tumor regression and enhance ROS-mediated cytotoxicity in BC models	Emerging platform for redox-engineered cancer therapy	Preclinical only—in vitro/in vivo models	[44,45]

**Table 4:** Context-dependent effects of antioxidant interventions in BC.

Issue	Earlier Perspective	Key Supporting Evidence	Practical Implication	References
**Antioxidant paradox**	Antioxidants generally considered protective against oxidative damage	Experimental models demonstrate that antioxidant supplementation can either suppress tumor growth or promote tumor progression depending on redox context (two of the cited studies are lung cancer and melanoma models, not breast cancer)	Antioxidant effects in cancer are strongly context-dependent	[16,195,196]
**Antioxidants and metastasis**	Antioxidants expected to reduce oxidative damage and inhibit tumor progression	Studies show that increased antioxidant capacity can enhance metastatic dissemination by improving tumor cell survival during circulation (melanoma model, not breast cancer)	Systemic antioxidant supplementation may promote metastasis in established tumors	[196]
**Loss of antioxidant enzymes**	Reduced antioxidant capacity assumed to impair tumor survival	GPx2 loss increases ROS levels, stabilizes HIF-1α and promotes metastatic progression in mammary tumor models	Redox imbalance can drive tumor progression via microenvironmental signaling	[16,197]
**Antioxidants during therapy**	Supplements frequently used to reduce chemotherapy toxicity	Clinical cohort analyses show increased recurrence risk when antioxidants are taken during chemotherapy	Antioxidant supplementation during cytotoxic therapy requires caution	[197]
**Dose-dependent effects**	Antioxidants viewed mainly as ROS scavengers	Redox-active compounds show biphasic behavior: antioxidant at low doses, pro-oxidant at higher doses	Careful dose optimization is critical in redox-targeted therapies	[40]
**Subcellular targeting**	Antioxidant strategies generally systemic	Mitochondria-targeted antioxidants suppress metastatic competence without interfering with therapy-induced ROS	Spatially targeted redox modulation may improve therapeutic specificity	[14,47]

## Data Availability

No new data were created or analyzed in this study. Data sharing is not applicable to this article.
